# Ancient DNA from *Palaeoloxodon naumanni* in Japan reveals early evolution of Eurasian *Palaeoloxodon*

**DOI:** 10.1016/j.isci.2025.114156

**Published:** 2025-11-21

**Authors:** Takahiro Segawa, Takahiro Yonezawa, Hiroshi Mori, Ayumi Akiyoshi, Asier Larramendi, Naoki Kohno

**Affiliations:** 1 Center for Life Science Research, University of Yamanashi, 1110 Shimokato, Chuo, Yamanashi, Japan; 2 Graduate School of Integrated Sciences for Life, Hiroshima University, 1-4-4 Kagamiyama, Higashi-Hiroshima, Hiroshima, Japan; 3 Genome Diversity Laboratory, National Institute of Genetics, Yata 1111, Mishima, Shizuoka, Japan; 4 Eofauna Scientific Research, Beloka 5, 2 eskubi, 20009 Donostia, Basque Country, Spain; 5 Department of Paleontology and Anthropology, National Museum of Nature and Science, Amakubo, Tsukuba, Ibaraki, Japan; 6 Graduate School of Life and Environmental Sciences, University of Tsukuba, Tennodai, Tsukuba, Ibaraki, Japan

**Keywords:** Zoology, Evolutionary biology, Paleobiology

## Abstract

*Palaeoloxodon*, the extinct genus of straight-tusked elephants, originated in Africa and dispersed across Eurasia. We analyzed ancient mitochondrial DNA of *Palaeoloxodon naumanni*, a species of the Japanese archipelago that went extinct during the late Late Pleistocene. The data indicate that *P. naumanni* forms a monophyletic group nested within the Eurasian-wide WE clade (defined by the Weimar-Ehringsdorf specimen and also represented in China) and represents an early-diverging lineage, with a split estimated at ∼1.05 Ma. Combined with fossil cranial morphology, the results support an early eastward spread of the plesiomorphic “Stuttgart morph” into East Asia, followed by continental predominance of the derived “*namadicus* morph”, while *P. naumanni* persisted as a relict in the Japanese archipelago under island isolation. These results enhance our understanding of the evolutionary trajectory of *Palaeoloxodon* and underscore the importance of ancient mitochondrial DNA analysis in shedding light on Pleistocene extinctions, with particular emphasis on the Japanese archipelago.

## Highlights

• Mitochondrial DNA recovered from *Palaeoloxodon naumanni* in Japan
• *P. naumanni* nests within the Eurasian-wide Weimar-Ehringsdorf clade
• Represents an early-diverging lineage dating to about 1.05 million years ago
• Evidence for early Stuttgart morph expansion, later replaced by namadicus morph

## Graphical abstract

**Figure:**
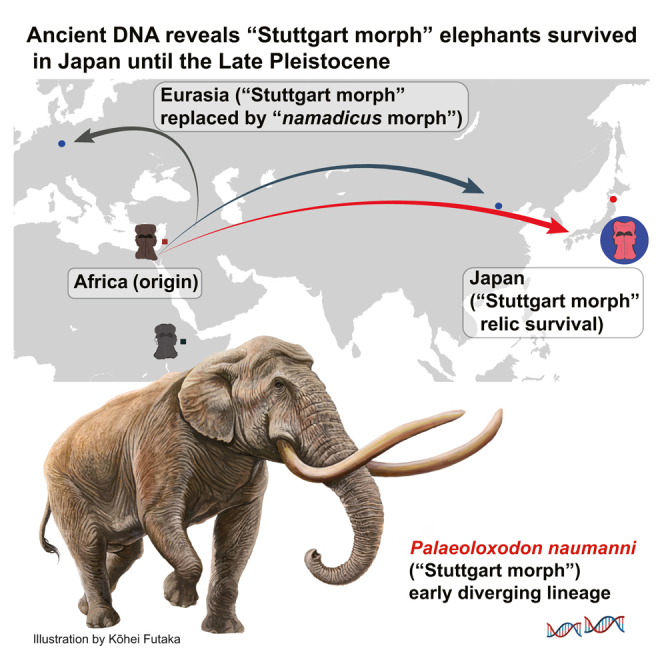

## Introduction

Elephantids, which were once widely distributed across the Northern Hemisphere as well as Africa, were prominent among megafauna and have played crucial roles as keystone species. The family Elephantidae originated in Africa and then diverged at least into eight genera, including *Stegotetrabelodon*, *Primelephas*, *Stegodibelodon*, *Loxodonta*, *Palaeoloxodon*, *Elephas*, *Stegoloxodon* and *Mammuthus*.^1^^,^^2^^,^^3^^,^^4^ *Elephas*, *Mammuthus*, and *Palaeoloxodon* extended their range into Eurasia, and fossil evidence suggests that *Palaeoloxodon* was the last of these three to migrate into Eurasia starting approximately 780,000 years ago (at the latest) and subsequently dispersed widely across Eurasia.^5^^,^^6^

The genus *Palaeoloxodon* was proposed by Matsumoto^7^ as a subgenus of *Loxodonta* for the Pleistocene Japanese species *Palaeoloxodon naumanni*, which was initially classified as *Loxodonta* (*Palaeoloxodon*) *namadicus naumanni*. However, others have synonymized it with *Elephas*,^1^ while many others have considered it a subgenus of *Elephas*.^8^^,^^9^^,^^10^ Nevertheless, accumulating morphological and genomic evidence indicates that *Palaeoloxodon* is most closely related to *Loxodonta* and, given its hybrid origin with further introgressions, should not be treated as a simple monophyletic clade distinct from *Elephas*. In particular, the mitochondrial lineages currently recognized (Neumark-Nord and the Eurasian-wide WE) fall within the forest elephants (*Loxodonta cyclotis*) clade (F clade), while nuclear genomic analyses point to this genus being the sister group of *Loxodonta*, subsequently established through hybridization with *L. cyclotis*.^6^^,^^11^^,^^12^

This long debate is largely owing to varying interpretations of morphological characteristics. The molars of *Palaeoloxodon* resemble those of Asian elephants (*Elephas*) in terms of occlusal plate count and frequency, and having finely folded enamel; however, their occlusal enamel patterns are more akin to those of African elephants (*Loxodonta*) in having lozenge-shaped enamel loops in the middle.^6^^,^^7^^,^^13^ Such morphological complexity has confounded the evolutionary relationship of *Palaeoloxodon* elephantids. Conversely, similarities in cranial traits with Asian elephants have led many researchers to believe that *Palaeoloxodon* is closely related to *Elephas*.^1^^,^^8^^,^^9^^,^^10^^,^^13^^,^^14^^,^^15^

Here, we refer to the Eurasian-wide WE clade as the lineage originally established by the Weimar-Ehringsdorf specimen (Germany) and later identified in Asia (CADG841, China). Recent phylogenetic analyses of genomic data have clarified aspects of the evolutionary history of mainland *Palaeoloxodon* in Europe.^11^^,^^12^^,^^16^ These studies revealed that *Palaeoloxodon* originated through several hybridization events based on three major components. The largest of these derived from the ancestor of African elephants (*Loxodonta*), followed by a significant gene flow from forest elephants (*L. cyclotis* [Matschie, 1900]^17^) and with a lesser genetic contribution from mammoths (*Mammuthus*).^11^^,^^12^ The entire mitochondrial genome of *P. antiquus* was replaced with that of *L. cyclotis* via interspecific hybridization, and currently two distinct mitochondrial lineages of *Palaeoloxodon*, namely, the Neumark-Nord (NN) and Weimar-Ehringsdorf (WE) clades, are identified within the *L. cyclotis* clade.^11^ Such discoveries have been instrumental in revising the evolution and taxonomy of *Palaeoloxodon* in Eurasia. The NN clade consists of *Palaeoloxodon* fossils discovered in Europe, whereas the Eurasian-wide WE clade encompasses elephantid fossils from European (Weimar-Ehringsdorf, Germany) and Asian (Yangyuan, Hebei, China) sites. However, the Weimar-Ehringsdorf fossil (bone fragment: KY499558) was not morphologically diagnostic and was identified as *P. antiquus* because of geochronological alignment as well as the geographic range of this species.^11^ Furthermore, the Yangyuan fossil (molar: CADG841), which was previously assigned as genus *Elephas* based on its morphology, was identified as *Palaeoloxodon* because of its maternal phylogenetic position.^16^ No *Loxodonta* fossils have been found outside Africa,^16^ suggesting that hybridization between *Palaeoloxodon* and forest elephants occurred in Africa before *Palaeoloxodon* expanded into Europe.^11^^,^^12^^,^^16^

The genetic contribution to European *Palaeoloxodon* is greater in West African forest elephants than in Central African forest elephants.^12^ This indicates that the hybridization between *Palaeoloxodon* and forest elephants occurred after the differentiation of the Central African and West African populations,^16^ which is estimated to have occurred between 0.60 and 0.46 Ma.^12^ On the other hand, the oldest fossil record of Eurasian *Palaeoloxodon* is dated to about 0.78 Ma.^5^ Thus, Lin et al.^16^ suggested that the lineage that entered into Eurasia from Africa by 0.78 Ma and the lineage that subsequently spread to Europe may have hybridized independently with the African elephants in a later event. However, as noted above, no morphological diagnoses exist among elephant fossils comprising the Eurasian-wide WE clade, and the taxonomy of the Eurasian-wide WE clade has not been confirmed. Since the Eurasian-wide WE clade corresponds to the lineage that first entered into Eurasia according to the hypothesis by Lin et al.,^16^ taxonomic confirmation of this clade is extremely important in order to test their hypothesis that a population that interbred with the forest elephants had spread throughout Eurasia as well as in Europe.

**Figure 1:**
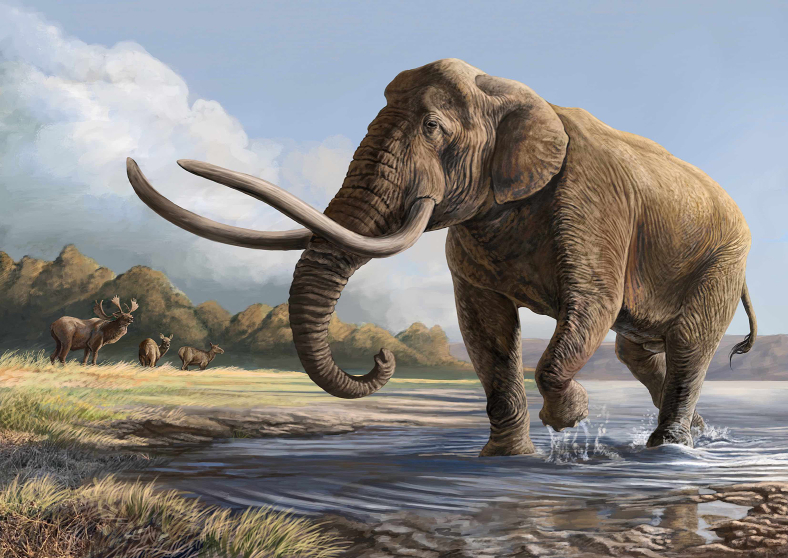
Biological Reconstruction of *Palaeoloxodon naumanni* This illustration is used with permission from the artist, Kōhei Futaka. The proportion of the head is based on a male skull described by Inuzuka.^19^ Curvature of the tusks is based on specimens reported in Inuzuka.^20^ Outline of the spine and proportion of the legs are based on skeletal remains of two female individuals depicted.^21^^,^^22^ Background herbivores are the giant deer *Sinomegaceros yabei* known as *Palaeoloxodon*-*Sinomegaceros* complex.^23^

Here we present the first DNA analysis of an extinct Japanese elephant, namely, *P. naumanni*,^18^ known as the Naumann’s elephant (Figure 1). The fossil record of *P. naumanni* comprises more than 2000 specimens unearthed from approximately 300 different sites across Japan, and the specimens represent the most prolifically recovered fossils in Japan.^21^ This extinct elephant species thrived in the late Middle to Late Pleistocene until 22,000 years ago.^21^^,^^24^ The abundance of recorded fossils underscores their once widespread existence and offers invaluable insight into the ecological and environmental conditions of Japan during the Late Pleistocene subepoch.

**Figure 2:**
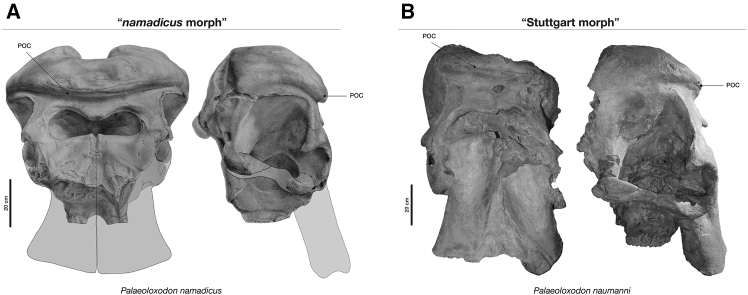
Comparison of “*namadicus*” and “Stuttgart” morphotypes Mature skulls of *P. namadicus* and *P. naumanni* showing the differences in the parieto-occipital crests (POC) between the two morphotypes. In the “*namadicus* morph”, the POC is much stronger and more anteriorly expanded, reducing the height of the frontals, compared with the “Stuttgart morph.” (A) NHMUK PV M3092 female skull, modified from Larramendi et al.^6^ (their Figure 1). (B) NMNS-PV 18700 male skull, modified from Takahashi^21^ (their Figure 4).

In Eurasia, several *Palaeoloxodon* species have been reported, i.e., *P. namadicus* (Falconer and Cautley, 1845),^14^ *P. antiquus* (Falconer and Cautley, 1847),^25^ *P. mnaidriensis* (Adams, 1874),^26^ *P. naumanni* (Makiyama, 1924),^18^ *P. turkmenicus* (Dubrovo, 1960),^27^ and *P. huaihoensis* (Liu, 1977),^28^ among others, with two primary cranial morphotypes recognized, namely, the “Stuttgart morph” and “*namadicus* morph” (Figure 2). Debate continues concerning whether these variations reflect distinct species,^9^^,^^19^ different growth stages, or simply individual variation within a single population.^6^^,^^29^ The former hypothesis may imply two migrations out of Africa, potentially corresponding to the distinct “Stuttgart morph” and “*namadicus* morph” populations. To date, DNA analysis of the *Palaeoloxodon* morphotypes has been limited, with genetic information available exclusively from “*namadicus* morph” specimens found in Sicily and Germany (i.e., *P.* cf. *mnaidriensis* and *P. antiquus*). Unfortunately, no genetic data have been obtained for the “Stuttgart morph” (i.e., *P. turkmenicus* and *P. naumanni*), creating a considerable gap in our understanding of the evolution of these prehistoric elephants. This absence of genetic information from the “Stuttgart morph” is an obstacle to studies of the evolutionary history of *Palaeoloxodon* and underscores the need for further research.

The Japanese archipelago has served as a refugium for wildlife, often preserving ancient lineages owing to its long isolation only with short-term connections to the continent during sea-level fluctuations in the Pleistocene epoch.^30^ Thus, the Japanese islands represent a key phylogeographic location for studying mammalian history. Accordingly, the study of ancient DNA of *P. naumanni*, in which the species is only known to present the “Stuttgart morph” in both mature females and males,^6^^,^^9^^,^^21^ is vital for piecing together the evolutionary puzzle of *Palaeoloxodon* species throughout Eurasia. Accordingly, we present the results of our analysis of ancient DNA of *P. naumanni*, which is recognized as a species exhibiting only the “Stuttgart morph” (Figure 1). These findings offer a crucial insight into the evolutionary history of *Palaeoloxodon* species across Eurasia.

## Results and discussion

### Phylogeny and divergence age of *P. naumanni*

Analysis of ancient DNA from *P. naumanni* revealed low mitochondrial DNA (mtDNA) preservation, with only a small amount of mtDNA sequences obtained. Sequences of mtDNA were not obtained from two of the four specimens (specimen NMNS-PV 22669 and AoPM 1975-5), and they were excluded from the analysis owing to an inadequate amount of mtDNA (Table S1). Partial mtDNA sequences were detected from the other two specimens (NMNS-PV 22670 and AoPM 1975-7), although the coverage was too low to reconstruct complete mitochondrial genomes from shotgun sequencing alone. To overcome this limitation, targeted enrichment using myBaits capture was performed. After removing PCR duplicates by sequence clustering, 7,770 mitochondrial read clusters were obtained from NMNS-PV 22670 and 6,137 read clusters from AoPM 1975-7. In both specimens, this enrichment enabled the reconstruction of draft mitochondrial genome sequences with an average depth of coverage per 1 kb window of approximately 10.7× and 8.2× (calculated by AMBER), respectively. The mitochondrial DNA of the two specimens exhibited signatures of postmortem damage (Figures S1 and S2). The average mitochondrial read lengths were 38.9 bp (NMNS-PV 22670) and 38.2 bp (AoPM 1975-7), and the minimum/maximum read lengths were 30/76 bp and 30/78 bp, respectively (Figures S3–S5). Note that the minimum read length of 30 bp does not reflect the shortest fragments recovered, but rather results from a uniform cutoff applied to filter out reads shorter than 30 bp. A phylogenetic tree could be constructed based on the draft mitochondrial sequences of the two *P. naumanni* specimens together with the complete mitochondrial genome sequences of other elephantid species (Figure S6). The two *P. naumanni* individuals were found to form a clade, and this clade further formed a monophyletic group along with the Eurasian-wide WE clade, which includes the Weimar-Ehringsdorf specimen from Germany and the CADG841 specimen from China, with *P. naumanni* being the earliest branch within this group. This phylogenetic relationship was strongly supported with high statistical confidence, indicating the robustness of the results (Figure S7; Table S2). The maximum likelihood tree (Figure S6) identifies two mitochondrial lineages in Eurasian *Palaeoloxodon*: (1) *P. naumanni* plus the Eurasian-wide WE clade and (2) a *namadicus*-associated lineage nested within *Loxodonta cyclotis* mitochondrial lineages. This pattern is consistent with at least one episode of mtDNA capture from *L. cyclotis*—most clearly in the *namadicus*-associated lineage—although mtDNA alone cannot distinguish a single event from multiple introgressions.

**Figure 3:**
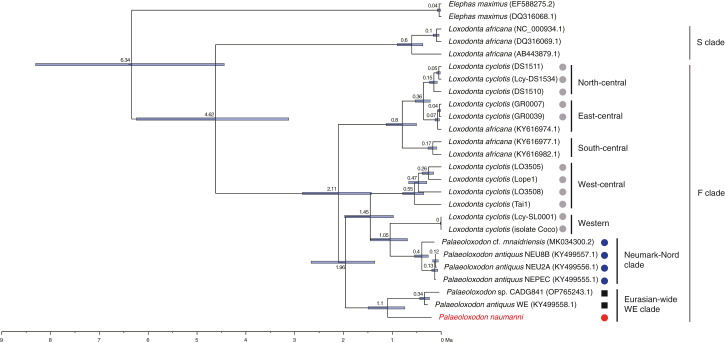
Divergence times among *Palaeoloxodon* species based on mitochondrial genome data analyzed by BEAST Nodal numbers indicate the median estimated divergence time in millions of years BP, and blue horizontal bars indicate the 95% highest posterior density (HPD) estimates for node age. In the present analysis, the sequences of the most recent common ancestor of the two *P. naumanni* individuals estimated by maximum likelihood method are used as representative of the *P. naumanni* lineage. Colored symbols denote morphotypes and affinities: red, “Stuttgart morph”; blue, “*namadicus* morph”; gray, lineages associated with *Loxodonta cyclotis* mtDNA; black, undetermined morphotype. For clarity, the “*P. naumanni* + Eurasian-wide WE” lineage and the “*namadicus*-associated” lineage are highlighted. Note that divergence ages represent maternal lineage history and are not direct dates of morphological innovation. Sub-clade designations for F clade follow Ishida et al. (2013).^31^

Inclusion of *P. naumanni* within the Eurasian-wide WE clade confirms that the Weimar-Ehringsdorf specimen from Germany and the CADG841 molar from China represent *Palaeoloxodon* that dispersed into Eurasia, rather than a forest elephant. The unusually long terminal branches of *P. naumanni* are attributable to ancient-DNA damage and PCR amplification bias in low-complexity libraries, which can inflate branch lengths and bias tip-based dating. Accordingly, given the monophyly of the two *P. naumanni* specimens, we reconstructed the ancestral mitochondrial genome sequence and estimated divergence from the remaining WE clade using this ancestral node (Figure 3). The divergence dates to approximately 1.05 Ma. To validate the ancestral-node reconstruction approach, we conducted robustness tests using UDG-treated *P. antiquus* mitogenomes from Neumark-Nord, evaluated by down-sampling (to 100–1,000 reads) and by introducing 1% artificial errors into both consensus sequences and raw reads. In both analyses, the divergence time between the Neumark-Nord individuals and the Sicilian individual remained stable at ∼0.4 Ma (Figures S8–S13; see STAR Methods), supporting the reliability of ancestral-node-based dating for error-prone ancient mtDNA.

The divergence times estimated in this study suggest that the most recent common ancestor of the Eurasian-wide WE clade is dated at approximately 1.05 Ma, suggesting that the hybridization event with *L. cyclotis* occurred sometime before this divergence, potentially any time between ∼1.93 Ma (divergence of the closest *L. cyclotis* lineage: Figure 3) and ∼1.05 Ma. Similarly, the potential hybridization event with *L. cyclotis* in the NN clade is inferred to have occurred sometime between ∼1.03 Ma (divergence of the closest *L. cyclotis* lineage) and ∼0.39 Ma (the tMRCA of the NN clade).

In addition, *Palaeoloxodon* exhibits both an ancestral African form and a derived Eurasian form. Which is actually the earliest species of *Palaeoloxodon* remains unresolved, although there is no doubt that it should have derived from one of the five subspecies described in the African Plio-Pleistocene “*Elephas recki*” complex (*E. recki brumpti; E. recki shungurensis; E. recki atavus; E. recki ileretensis*, and *E. recki recki*).^6^^,^^32^ These wastebasket taxa may include not only different species but also genera.^6^^,^^32^ Based on craniodental apomorphies, the possible earliest member of *Palaeoloxodon* could be represented by *Elephas recki atavus*,^8^^,^^9^ whose fossils can be traced from the Lower Pleistocene deposits of the Ethiopian Shungura Formation and date to approximately 2.34–1.90 Ma.^4^ However, there is not a consolidated consensus yet.^6^^,^^33^ The only subspecies that exhibit unambiguous craniodental apomorphies of *Palaeoloxodon,* including noticeable development of the parieto-occipital crest (POC) and distally and laterally flared premaxillaries, is *E. recki* *recki* represented by BOU-VP-3/131 cranium discovered in Bouri, Ethiopia (Daka Member) dating approximately 1 Ma.^6^^,^^9^ However, the somewhat earlier middle Early Pleistocene *E. recki ilerentensis* (i.e., KNM-ER 799 juvenile cranium) may belong to *Palaeoloxodon* as well.^6^

Among African *Palaeoloxodon*, the morphological features found in *E. recki* *recki* are derived traits shared with Eurasian *Palaeoloxodon*, and the date of appearance of these derived morphotypes of *Palaeoloxodon* (about 1 Ma) overlaps with the hypothesized time frame of hybridization with forest elephants in both the Eurasian-wide WE clade and the NN clade. These dates also align with an apparent discontinuity in the morphological evolution, transitioning from straight premaxillaries to laterally flared premaxillaries.

**Figure 4:**
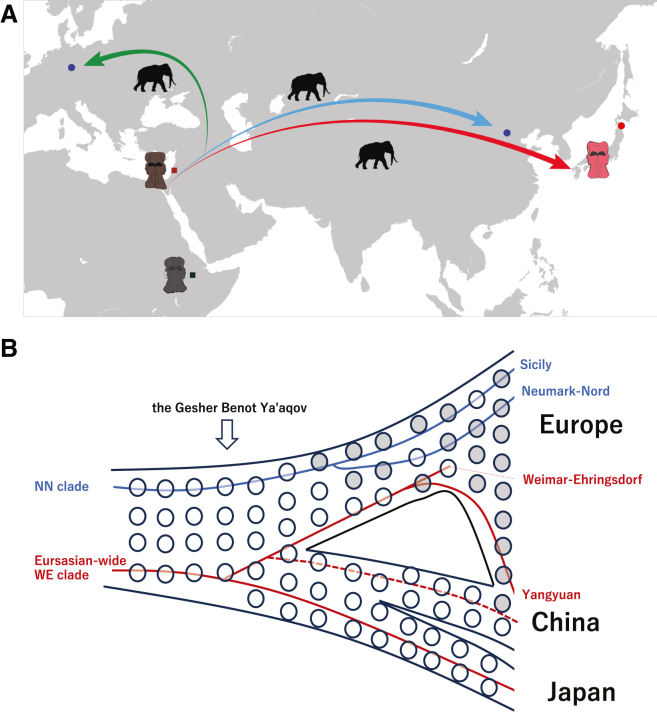
Conceptual evolutionary scenario for Eurasian *Palaeoloxodon* (A) Schematic diagram of the conceptual hypothesized migration of *Palaeoloxodon* into Eurasia. Black square dot represents the oldest known Eurasian form of *Palaeoloxodon* with unambiguous characters discovered at Daka, Ethiopia (approximately 1 Ma). Dark-red square dot indicates the oldest known Eurasian fossil from Gesher Benot Ya’aqov (0.78 Ma). Blue circle dots indicate the *Palaeoloxodon* fossils from the Eurasian-wide WE clade from Europe (Weimar-Ehringsdorf, Germany: 0.216–0.25 Ma) and Asia (Yangyuan, Hebei, China: >0.0503 Ma) reported by previous studies. Red circle dot indicates the fossils of *P. naumanni* used in this study (Aomori, Japan: oldest fossil is 49,880 cal BP). This panel is conceptual; localities and ages are data based, whereas arrows indicate hypothesized dispersal and replacement within the WE clade. Arrows depict a conceptual diagram of phylogeography within the Eurasian-wide WE clade. The red arrow represents an early-divergent lineage within this clade (*P. naumanni* clade) that spread into eastern Eurasia shortly after *Palaeoloxodon* entered Eurasia. The blue arrow represents the Chinese *Palaeoloxodon* lineage (Yangyuan), which diverged later from the *P. antiquus* lineage within the WE clade in Europe (indicated by the green arrow). (B) Phylogeographic relationships among the maternal lineages of Eurasian *Palaeoloxodon* and associated phenotypic polymorphisms (open circles: “Stuttgart morph”; gray-shaded circles: “*namadicus* morph”). Eurasian *Palaeoloxodon* has two primary maternal lineages, potentially due to ancestral polymorphism (single hybridization hypothesis) or independent hybridization events. The blue line represents the NN clade, while the red line represents the Eurasian-wide WE clade. The “*namadicus* morph” likely originated in western Eurasia during the Middle Pleistocene, and subsequently spread to eastern Eurasia. The divergence of *P. antiquus* in the WE clade in Europe and Chinese *Palaeoloxodon* (CADG841) occurred after the emergence of the “*namadicus* morph” phenotype. Therefore, the maternal lineage of the Chinese *Palaeoloxodon* (CADG841) likely reflects gene flow from Western Eurasia into Eastern Eurasia. An alternative maternal scenario for the Chinese specimen is also indicated (red path), in which the spread of the “*namadicus* morph” into Asia is decoupled from WE movement from Europe to Asia.

*Palaeoloxodon* ancestors are thought to have migrated into Eurasia post hybridization,^12^ yet the timing of their subsequent expansion into East Asia remains uncertain, potentially dating to the Middle Pleistocene.^5^ Results from our present analysis suggest that the mitochondrial lineage of *P. naumanni* diverged from the Eurasian-wide WE clade at approximately 1.05 Ma, close to the age of the oldest Eurasian fossil at the GBY site (0.78 Ma). This implies that the population of *Palaeoloxodon* spread throughout Eurasia, including East Asia, ensuing shortly after their migration from Africa, and the *P. naumanni* lineage can be interpreted as an early branch within the Eurasian radiation. Together, these results motivate the conceptual evolutionary scenario summarized in Figure 4, which integrates maternal lineages and dispersal routes.

### “Stuttgart” and “*namadicus*” morphotypes of *Palaeoloxodon*

Various Asian *Palaeoloxodon* species have been identified, such as *P. naumanni* and *P. huaihoensis* as well as the European *P. antiquus* and *P.* cf. *mnaidriensis,* Central Asian *P. turkmenicus*, and Indian *P. namadicus*, and they all can be categorized into two morphotypes, namely, “Stuttgart morph” and “*namadicus* morph”, based on skull morphology; still, no consensus of their categorization has been achieved. The “Stuttgart morph” and “*namadicus* morph” concepts were proposed by Saegusa and Gilbert in 2008^9^ to differentiate two cranial morphotypes within the genus *Palaeoloxodon*. The former is characterized by weak development of the POC, positioned high on the frontals of the cranium, exemplified by ontogenetically immature *P. antiquus* skulls such as SMNS 32888 from Bad Cannstatt in Stuttgart, Germany, as well as adult specimens of *P. turkmenicus* from Central Asia and *P. naumanni* from Japan. In contrast, the “*namadicus* morph” exhibits a strongly developed POC that extends forward and overhangs much of the frontals, approaching the external nasal aperture, a feature typical of the Indian *P. namadicus*.^6^ Each of these morphotypes, as recognized in both Asia and Europe, is suggested to represent either distinct taxonomic differences^9^ or varying developmental stages within or among species.^6^^,^^29^ Nevertheless, the previous idea that the “Stuttgart morph” was present in European deposits for mainland Europe *P. antiquus* has been recently clarified. Larramendi et al.^6^ observed that specimens with a weakly developed POC (the “Stuttgart morph”) were associated with ontogenetically young individuals. In contrast, fully grown individuals from both northern and southern Europe all exhibited a well-developed POC, indicating that only the “*namadicus* morph” is present in mainland Europe *Palaeoloxodon*. However, there is an exception, the skull (152-E9) from Neumark-Nord 1.^6^^,^^29^ Nonetheless, this skull displays a significant oval-shaped opening on the frontals, an injury sustained earlier in life,^29^ and the absence of a well-developed POC could be attributed to this pathology.^6^ Nevertheless, this skull also exhibits a highly elevated skull—a plesiomorphic condition in *Palaeoloxodon*—similar to *P. turkmenicus* and *P. naumanni*, but distinct from the *P. antiquus* specimens from southern Europe,^6^^,^^19^^,^^27^ although it is difficult to ascertain to what extent the frontal injury contributed to this shape.

Because complete skull specimens are necessary to distinguish Stuttgart and *namadicus* morphotypes, it is challenging to pinpoint the emergence of these differences within the context of the incomplete fossil record. In this regard, a cranium of an elephantid possibly having strong POC was identified by Sonakia and Biswas^34^ as *P. namadicus* (i.e., “*namadicus* morph”) from the lower Middle Pleistocene Dhansi Formation (ca. 2.4–0.7 Ma) in Narmada, India,^34^ and it was referred by Larramendi et al.^6^ as the oldest known record of *P. namadicus*. However, the actual locality and source rock of the specimen has not been mentioned clearly in the study by the former authors, and the reliable record of this species in India is considered at present to be the specimens from the late Middle to Late Pleistocene age (ca. 160–10 Ka) from the lower unit of the Surajkund Formation to the Baneta Formation.^35^^,^^36^^,^^37^ Accordingly, the earliest reliable fossil of the “*namadicus* morph” dates back to Marine Isotope Stage 12 (MIS 12) in Marathousa 1, Greece, approximately 0.48–0.42 million years ago (Ma).^38^ Nevertheless, earlier occurrences of this morphotype cannot be ruled out until an adult cranium of a previous date is found. Current molecular evidence acquired from the “*namadicus* morph” specimens is limited to specimen GP4 excavated from Puntali Cave in Sicily, which belongs to the NN clade. In addition, all specimens from Neumark-Nord, Germany, that were used by Meyer et al.^11^ also belong to the NN clade. All *P. antiquus* adult specimens from Neumark-Nord have the *namadicus* morphotype except for specimen 152-E9 as explained above.^6^^,^^29^ Another specimen, 168-E30, is the same individual as HK 2007:25:280 = E15.1.9 [NEPEC], and although it is not entirely clear, molar specimen HK 2007:25.285,117 [NEU2A] probably belonged to 175-E 23B. These individuals have a prominent POC. The time of the most recent common ancestor of the NN clade was estimated approximately 0.4 Ma. On the other hand, the weakly developed POC of the“Stuttgart morph” is thought to be a plesiomorphic condition.^6^^,^^9^ Because only “Stuttgart morph” is known to be present in adult *P. naumanni*, our present results constitute the first molecular data from a definitive Stuttgart morphotype *Palaeoloxodon*, confirming its inclusion in the Eurasian-wide WE clade.

Lin et al.^16^ suggested that the two Eurasian *Palaeoloxodon* clades resulted from independent hybridization events. The morphological differences observed between each mitochondrial clade may reflect differences in how alleles from the two ancestral species became fixed within each population. Further research is imperative to clarify the exact relationships among these clades and morphotypes. Moreover, Lin et al.^16^ assumed two independent hybridization events based on the divergence time between Central African and West African populations of forest elephants, an estimate derived under the assumption of complete genetic isolation between those two populations.^39^ However, considering the geographic distribution of forest elephants, it is more plausible that gene flow occurred between the Central and West African populations. If so, the true divergence time between them would be much older than previously estimated. Consequently, the possibility that Eurasian *Palaeoloxodon* originated from a single hybridization event with forest elephants cannot be excluded. To more accurately determine the frequency and timing of these hybridization events, nuclear genome analysis of Asian *Palaeoloxodon* individuals will be required. Our data suggest that *P. naumanni* represents an earlier diverging lineage within Eurasian *Palaeoloxodon*, and at least the most basal lineage within the Eurasian-wide WE clade. Its divergence dates to approximately 1.05 Ma, preceding the first appearance of the “*namadicus* morph” in Eurasia (0.406–0.48 Ma) (Figure 4). Considering that *P. naumanni* consistently exhibits the Stuttgart morphotype in both mature females and males, it is plausible that this lineage became genetically distinct from other Eurasian populations in its early history. The inclusion of the also rather plesiomorphic Central Asian Middle Pleistocene “Stuttgart morph” *P. turkmenicus*—which exhibits the weak POC development among *Palaeoloxodon* species, strikingly similar to that of the Neumark-Nord 152-E9 specimen,^6^ although the latter appears to be pathological—within the Eurasian-wide WE clade is difficult to ascertain in the absence of aDNA from this enigmatic species. Owing to its morphological similarities to *P. naumanni*, this possibility cannot be entirely excluded. Nevertheless, it should be considered that the distinctive morphology of *P. turkmenicus* may alternatively have arisen through an atavistic mutation derived from a *P. antiquus*-like ancestor^40^ that may have belonged to a different clade.

During the Pleistocene, the Japanese archipelago served repeatedly as a refugium for various species, allowing ancient lineages to survive as relicts owing to the long isolation of the island only with short-term connections to the continent because of periodic fluctuations of sea levels.^41^^,^^42^^,^^43^^,^^44^ *P. naumanni* is thus regarded as a relict population that survived in Japan until the Late Pleistocene, exhibiting the plesiomorphic characters of the Eurasian *Palaeoloxodon* including a weakly developed POC, downturned lateral margins of the nasals, and a very high cranium.^6^ Although *P. naumanni* was identified as having the Stuttgart morphotype, the “*namadicus* morph” is commonly observed in continental East Asia. In fact, a solitary complete cranial fossil from the Late Pleistocene Nihewan, Hebei, China, has been unambiguously classified as “*namadicus* morph”.^6^^,^^45^^,^^46^ The paucity of intact skulls in the fossil record does not allow for a definitive conclusion concerning the diversity of morphotypes present in Eurasia, leaving it unclear if the *namadicus* morphotype was exclusive in ancient times or if Stuttgart and *namadicus* morphotypes coexisted. Our results provide insights, specifically that *P. naumanni* of the Japanese archipelago was the most anciently diverged Eurasian lineage. Our results also suggest an early dispersal of the “Stuttgart morph” into East Asia, which was later supplanted by the population represented by the *namadicus* morphotype (Figure 4).

Saegusa and Gilbert,^9^ through their morphological analysis, hypothesized that *P. naumanni* may represent the ancestral lineage of Eurasian *Palaeoloxodon* and that it persisted as a relict population within the Japanese archipelago without being replaced by a later population having the “*namadicus* morph” due to geographical isolation. Given the time scales of population structure in elephantids,^12^ episodes of gene flow among Eurasian *Palaeoloxodon* species from the late Early to Middle Pleistocene are plausible. The derived “*namadicus* morph”, which is expressed in sexually mature individuals, emerged in Europe before MIS 12 (0.48 Ma) and was nearly fixed in the European population (i.e., *P. antiquus*). The “*namadicus* morph” further spread into Asia in the Late Pleistocene, as seen in the complete skull of the Nihewan (China) specimen (i.e., IVPP 4443). This specimen exhibits shared derived characters (“*namadicus* morph”) with those of *P. antiquus* in Europe. On the other hand, as mentioned above, *P. naumanni*, which migrated to the Japanese archipelago during the Middle Pleistocene, avoided invasion by Eurasian continental populations owing to its geographical isolation in the Japanese islands. *P. naumanni* survived until the late Pleistocene, with the most recent fossil record at approximately 22,000 years ago,^24^ indicating that this species became extinct around that time. There is an expectation that comprehensive analyses of the *P. naumanni* nuclear genome, i.e., utilizing numerous specimens, will enhance our understanding of the evolutionary history of Eurasian *Palaeoloxodon* and the factors that contributed to the extinction of *P. naumanni*. However, due to the low depth of coverage of the sequence data in this study, the two mitochondrial genome sequences contain approximately 10% of unknown nucleotide sites, and nuclear DNA could not be reliably obtained from these specimens. Consequently, the detailed evolutionary history of *P. naumanni* remains unclear. Future advancements in technical approaches, including sediment-derived ancient DNA analysis and other high-resolution genomic methods, will be crucial for overcoming these limitations and providing deeper insights into the species’ evolutionary history and the factors leading to its extinction.

### Limitations of the study

We recovered capture-enriched draft mitochondrial genomes from two *P. naumanni* individuals (NMNS-PV 22670 and AoPM 1975-7); nuclear DNA could not be reliably obtained. Given documented introgression in *Palaeoloxodon*, mtDNA alone cannot unambiguously resolve the number or timing of hybridization events or the genome-wide relationships among Eurasian clades. Divergence-time estimates rely on an ancestral-node reconstruction applied to low-coverage mitogenomes assembled from reads that show characteristic postmortem damage and should be interpreted with caution. Our sampling is geographically restricted to the Japanese archipelago and lacks confirmed continental “Stuttgart morph” genomes; broader sampling and nuclear genomic data will be needed to test demographic scenarios and refine timing.

## Resource availability

### Lead contact

Further information on materials, datasets, and protocols should be directed to and will be fulfilled by the lead contact, Takahiro Segawa (tsegawa@yamanashi.ac.jp).

### Materials availability

This study did not generate new unique reagents. Fossil specimens are curated at the National Museum of Nature and Science (Japan) and the Aomori Prefectural Museum (Japan) under the catalog numbers listed in the key resources table; access is subject to each repository’s policies.

### Data and code availability

• Data: Raw sequencing reads are deposited in the DDBJ Sequence Read Archive (DRA) under accession numbers DRR751097 (NMNS-PV 22670) and DRR751098 (AoPM 1975-7), with automatic mirrors at ENA and NCBI SRA. Mitochondrial consensus sequences are available in DDBJ/ENA/GenBank under LC897757 (NMNS-PV 22670) and LC897756 (AoPM 1975-7). The project-level record is registered under DDBJ BioProject PRJDB17056. Dereplicated mitochondrial read clusters, including the trimmed versions used for consensus reconstruction, are publicly available at http://palaeo.nig.ac.jp/Resources/24Nau/.
• Code: No new code was generated in this study. All software used is listed in the key resources table.
• Other items: Any additional information required to reanalyze the data reported in this paper is available from the lead contact upon request.

## Acknowledgments

We thank Drs. Takashi Shimaguchi from the Aomori Prefectural Museum, Japan, and Yuri Kimura from the National Museum of Nature and Science for granting permission to use the specimens. We also express our gratitude to Drs. Fuyuki Tokanai from the Yamagata University and Ayako Kohno from the University of Tokyo for conducting the radiocarbon dating analysis of specimens NMNS-PV 22669 and 22670. This study was supported by Grants-in-Aid for Scientific Research (no. 20K20942, 23KK0062, 25K01110 and JP221S0002) from the 10.13039/501100001691Japan Society for the Promotion of Science. The research was conducted with reference to knowledge of the morphological analysis of *Palaeoloxodon*, a discipline in which we were guided by the late Dr. Haruo Saegusa. We express our deepest gratitude for Dr. Saegusa’s mentorship. Computations were partially performed using the supercomputer of the National Institute of Genetics at the Research Organization of Information and Systems. We also sincerely thank the anonymous reviewers for their valuable comments, which greatly improved the quality of the manuscript.

## Author contributions

T.S., T.Y., and N.K. designed the project. A.A. and T.S. supplied the genomic sequences. T.S., H.M., and T.Y. analyzed the sequencing data. T.S., T.Y., H.M., A.L., and N.K. wrote the manuscript. All authors gave final approval for publication.

## Declaration of interests

The authors declare no competing interests.

## STAR★Methods

### Key resources table

**Table:** 

REAGENT or RESOURCE	SOURCE	IDENTIFIER
**Biological samples**		

*Palaeoloxodon naumanni* molars from Shiriya Limestone quarry	National Museum of Nature and Science, Japan	NMNS-PV 22669
*Palaeoloxodon naumanni* molars from Shiriya Limestone quarry	National Museum of Nature and Science, Japan	NMNS-PV 22670
*Palaeoloxodon naumanni* molars from Shiriya Limestone quarry	AoPM (Aomori Prefectural Museum)	AoPM 1975-5
*Palaeoloxodon naumanni* molars from Shiriya Limestone quarry	AoPM (Aomori Prefectural Museum)	AoPM 1975-7

**Chemicals, peptides, and recombinant proteins**		

N-Lauroylsarcosine (sodium salt)	Sigma Aldrich	Cat# 8147150100
UltraPure 0.5 M EDTA, pH 8.0	Invitrogen	Cat# 15575-020
Sodium acetate solution 3M, pH 5.2	Sigma-Aldrich	Cat# S7899
Proteinase K, recombinant, PCR grade	Roche	Cat# 3115844001
EB Buffer	QIAGEN	Cat# 19086
PB Buffer	QIAGEN	Cat# 19066

**Critical commercial assays**		

NEBNext DNA Library Prep Master Mix Set for 454	NEB	Cat# E6070
End Repair Module	NEB	Cat# E6050
MinElute PCR Purification kit	QIAGEN	Cat# 28006
NucleoSpin Gel and PCR Clean-up kit	Macherey-Nagel	Cat# 740609
KAPA HiFi HotStart Uracil+ ReadyMix	Roche	Cat# KK2802
Qubit dsDNA HS Assay Kit	Thermo Fisher Scientific	Cat# Q32854
High Sensitivity DNA Kit	Agilent	Cat# 5067-4626

**Deposited data**		

Raw genomic data and mitochondrial genome sequences	This paper	BioProject: PRJDB17056
Raw reads	This paper	DDBJ DRA: DRR751097 and DRR751098
Mitochondrial genomes (consensus)	This paper	INSDC DDBJ/ENA/GenBank: LC897757 (NMNS-PV 22670); LC897756 (AoPM 1975-7)
Mitochondrial read clusters	This paper	http://palaeo.nig.ac.jp/Resources/24Nau/

**Software and algorithms**		

fastp (v0.20)	Chen et al.^47^	https://github.com/OpenGene/fastp
BWA aln (v0.7.17)	Li and Durbin^48^	https://github.com/lh3/bwa
VSEARCH	Rognes et al.^49^	https://github.com/torognes/vsearch
IGV	Thorvaldsdóttir et al.^50^	https://software.broadinstitute.org/software/igv/
mapDamage (v2.0.9)	Jónsson et al.^51^	https://github.com/ginolhac/mapDamage
AMBER	Dolenz et al.^52^	https://github.com/tvandervalk/AMBER
MAFFT	Katoh and Standley^53^	https://mafft.cbrc.jp/alignment/software/
IQ-TREE (v2.1.3)	Nguyen et al.^54^	http://www.iqtree.org/
PAML (v4.9)	Yang^55^	http://abacus.gene.ucl.ac.uk/software/paml.html
BEAST (v1.10.4)	Suchard et al.^56^	https://github.com/beast-dev/beast-mcmc/releases/tag/v1.10.4

**Other**		

Agilent 2100 Bioanalyzer	Agilent	Model G2939A
Illumina HiSeq 2500/4000	Illumina	HiSeq 2500/HiSeq 4000
Illumina NovaSeq 6000	Illumina	NovaSeq 6000
Micro drill	Nakanishi	Emax Evolution drill

### Experimental model and subject details

The *Palaeoloxodon naumanni* specimens NMNS-PV 22669 and NMNS-PV 22670 are stored at the National Museum of Nature and Science, Japan. The other *P. naumanni* specimens AoPM 1975-5 and AoPM 1975-7 are stored at Aomori Prefectural Museum, Japan. All specimens were excavated in April 1970 from the Shiriya Limestone quarry, Higashidori Village, Aomori Prefecture, Japan. Radiocarbon dating indicates ages ranging from ∼49,200 to ∼27,700 cal BP, corresponding to the late MIS 3 to early MIS 2. See method details for further information.

### Method details

#### Sample descriptions

As mentioned above, the skull material with molars of *P. naumanni* collected from the Japanese Islands^9^^,^^21^^,^^22^^,^^57^^,^^58^ consistently show the “Stuttgart” morph and, so far, no exceptions have been reported. This supports the conclusion that only the “Stuttgart” morph is present in *P. naumanni* in the Japanese Islands, reinforcing the consistency of the observed morphotypes and ruling out the presence of the “*namadicus*” morph in this context. Accordingly, we include the molars without skulls but identical to those with skulls as *P. naumanni*. In this context, the *P. naumanni* specimens analyzed in this study were excavated in April 1970 from the limestone ore transportation tunnel at the Shiriya Limestone quarry, operated by the Nittetsu Mining Co., in Shikkari, Higashidori Village, Shimokita Peninsula, Aomori Prefecture, Japan. Although the exact details of their initial discovery and excavation remain undocumented, the fossils were dispersed among several institutions^59^: Kyoto University housed one incisor, seven molars, and one right mandibular fragment; Shikkari Elementary School in Higashidori Village kept one incisor and two molars; and six molars were privately owned. In total, at least fifteen molars were reported. Five of these teeth were later described in detail in previous studies.^60^^,^^61^ Two of the privately owned specimens were eventually transferred to the National Museum of Nature and Science (NMNS) and cataloged as NMNS-PV 22669 and 22670 (Figure S14). The Kyoto University collection was also transferred to AoPM after detailed descriptions and ^14^C dating as reported.^21^^,^^59^ According to their findings, two samples (AoPM 1975-5 and AoPM 1975-7) had ^14^C ages of 23,570 ± 130 BP (time before present) and 29,920 ± 120 BP, which correspond to 27,747 ± 104 and 34,405 ± 110 cal BP, respectively, using the IntCal20 calibration curve. These dates suggest that the specimens belonged to the late MIS 3 to early MIS 2 period. Based on a ^14^C analysis, the calendar-year age of NMNS-PV 22669 was estimated to be 49,880 cal BP, and that of NMNS-PV 22670 was 49,218 cal BP (Drs. Fuyuki Tokanai and Ayako Kohno, personal communication).

#### Extraction of DNA from ancient samples, genome library construction, and sequencing

Prior to genome library amplification, all ancient DNA work was conducted in a dedicated clean room for ancient DNA at the University of Yamanashi and National Institute of Polar Research, Tokyo, Japan. From each of the four specimens, 500 mg sample of ivory powder was extracted using an Emax Evolution drill (Nakanishi, Japan). We extracted 100–300 mg of molars per DNA extraction session. We followed the DNA extraction procedure based on silica pellets in solution as described previously.^62^ Each sample was pre-digested via incubation at 42°C for 10 min in 1 mL digestion buffer (0.5 M EDTA, 0.2 mg/mL proteinase K, 0.5% N-lauroylsarcosine (sodium salt)). After centrifugation for 2 min at 2000 × *g*, the supernatant (digested material) was removed, and the pellet was incubated for 36 h at 42°C in 3 mL fresh digestion buffer. After centrifugation for 2 min at 2000 × *g*, the supernatant was incubated for 3 h at room temperature with 100 μL silica beads and 40 mL binding buffer (Qiagen PB buffer; 25 mM NaCl, 87 mM sodium acetate). After centrifugation for 5 min at 2000 × *g*, the supernatant was discarded, and the pellet was washed twice with 1 mL of 80% ethanol before eluting the DNA with 100 μL elution buffer (Qiagen). We also extracted DNA using silica column-based protocol optimized to ultrashort DNA fragments.^63^

Double-stranded DNA libraries were prepared using the NEBNext Quick DNA Library Prep Master Mix Set for 454 (New England BioLabs). Libraries were prepared with 20 μL DNA extract or damage-repaired DNA extract without DNA fragmentation. Illumina sequencing adapters were added to the end-repair reaction at a final concentration of 0.25 μM together with 1 U Quick T4 DNA ligase. DNA fragments in each reaction were purified using the MinElute PCR Purification kit and eluted with 20 μL EB buffer (included with the kit) at 37°C for 10 min. PCR amplification of each genomic library was carried out in a laboratory designed specifically for work with ancient DNA, and the amplified products were transferred to a thermal cycler in a conventional laboratory, which was physically separated. Three microlitres of the eluate were subjected to PCR amplification in 50 μL of reaction mixture containing KAPA HiFi HotStart Uracil+ReadyMix (Kapa Biosystems) and 0.3 μM Dual Index primers of NEBNext Multiplex Oligos for Illumina (NEB). PCR conditions consisted of an initial denaturation for 45 s at 98°C, 12–14 cycles of 15 s at 98°C, 30 s at 65°C and 30 s at 72°C, and a final extension for 1 min at 72°C. Three independent reaction mixtures were combined after PCR and purified using the MinElute PCR Purification kit. Single-stranded DNA libraries were also prepared following the protocol described by Gansauge et al.^64^ The PCR products were separated by 3% agarose gel electrophoresis and purified using a NucleoSpin Gel and PCR Clean-up kit (Macherey-Nagel).

Hybridization capture was performed on single-stranded DNA libraries using the myBaits Expert Mito Kit (Daicel Arbor Biosciences), targeting *Loxodonta africana* (NC_000934). Capture experiments followed the High Sensitivity Protocol included in the myBaits Manual (version 5.03), with minor modifications. Up to 1,000 ng of single-stranded library DNA was combined with blocking and hybridization reagents as specified in the manufacturer’s protocol. Both the DNA–blocking mixture and hybridization mix were denatured at 95°C for 5 min and pre-hybridized at 55°C for 5 min. Hybridization was conducted at 55°C for 20 h in a thermocycler. Captured libraries were washed and eluted according to the myBaits protocol. Half of the eluate was amplified using the KAPA HiFi HotStart Ready Mix under the following conditions: 98°C for 2 min; 14 cycles of 98°C for 20 s, 60°C for 30 s, and 72°C for 45 s; followed by 72°C for 5 min. A second round of hybridization capture was performed under the same conditions, using a reduced bait concentration. After purification, the enriched libraries were amplified by PCR for 8 cycles. The resulting products were separated on a 3% agarose gel and purified using the NucleoSpin Gel and PCR Clean-up Kit.

The quality of the library and the library size were assessed with an Agilent 2100 Bioanalyzer using the High Sensitivity DNA Kit (Agilent). Sequencing was carried out with an Illumina HiSeq 2500 and NovaSeq 6000 at National Institute of Genetics and an Illumina HiSeq 4000 at Macrogen, Japan. This generated 4,486,224,201 sequencing reads. The sequencing information is listed in Table S1.

#### Bioinformatics

Sequenced reads were quality filtered using fastp version 0.20^47^ with the parameter “-n 1 -L 30 -x”. High-quality reads (>30 nt) were mapped to the mitochondrial genome sequences of three elephant species (*Elephas maximus* NC_005129, *Loxodonta africana* AB443879, and *L. cyclotis* NC_020759) using BWA aln version 0.7.17^48^ with the parameter “-l 1024 -n 0.01 -o 2”. The reads that mapped to the mitochondrial genomes were subjected to the VSEARCH^49^ dereplication and clustering with parameters “--derep_fulllength --sizeout --minseqlength 30” and “--cluster_size --id 0.93 --iddef 0 --sizeout --strand both --minseqlength 30 --qmask none” to remove PCR duplicates. All NMNS-PV 22670 and AoPM 1975-7 mitochondrial read clusters were BLASTN searched with E-value <100 against the NCBI nt database and the read clusters that were top-hit to the elephant taxa were used. These read clusters were remapped to the mitochondrial genome sequence of *L. africana* using BWA aln version 0.7.17 with the parameter “-l 1024 -n 0.01 -o 2”. Using the read cluster-mapping results to the mitochondrial genome sequence, a consensus sequence based on the mitochondrial genomes of NMNS-PV 22670 and AoPM 1975-7 was reconstructed using IGV,^50^ respectively. To minimize the effect of postmortem nucleotide substitutions near the 5′ and 3′ ends of the read for the consensus mitochondrial genome sequence reconstruction, we trimmed 3 bp from the 5′ and 3′ ends of the read clusters. The DNA damage pattern of each sample was observed using MapDamage version 2.0.9^51^ with reference to the mitochondrial genome sequence of *L. africana* (AB443879.1) (Figures S1 and S2). The read mapping quality of NMNS-PV 22670 and AoPM 1975-7 was summarized using AMBER.^52^

#### Phylogenetic analysis based on the complete mtDNA sequence data

The analysis included two draft mitochondrial genome sequences of *P. naumanni* determined in this study and 26 complete mitochondrial genomes of other elephantid species reported previously (see Figure 3 for accession numbers of the 26 mitochondrial genomes downloaded from NCBI). The 13 mitochondrial protein-coding genes, 22 tRNAs, and two rRNAs as well as the D-loop were aligned with MAFFT.^53^ Start and stop codons as well as the overlapping regions between *ATP8* and *ATP6*, *ND4L* and *ND4*, and *ND5* and *ND6* were excluded. Protein-coding genes and RNAs (tRNAs and rRNAs) were concatenated, respectively. As for the regions encoded in the L strand such as *ND6*, the complementary sequences were used to account for nucleotide composition biases. Subsequently, protein-coding genes (11,208 bp), RNAs (4031 bp), and the D-loop (688 bp) were further concatenated, and the final alignment comprised 15,927 bp. Of these, the number of unambiguous bases was 14,301 bp for NMNS-PV 22670 and 14,569 bp for AoPM 1975-7. A maximum-likelihood (ML) tree was inferred using IQ-TREE ver. 2.1.3^54^ with the GTR+I+Γ model selected by the Bayesian Information Criterion, and 1,000 standard bootstrap replicates were used to estimate nodal support. Taking account of the different tempo and mode of the nucleotide substitutions, the alignment was separated into five partitions (the first, the second, and the third codon positions of the protein-coding genes, RNAs, and D loop).

#### Estimating divergence times among *P. naumanni*

The ML tree including two draft mitochondrial genome sequences determined in this study revealed the extremely long terminal branches of *P. naumanni* (Figure S6). These long branches were potentially a consequence of damage to the ancient DNA, making it difficult to estimate divergence times even under the independent rate model of the relaxed clock, which allows rates to vary throughout the tree. Because these kinds of sequence errors mainly affect the terminal branches, the ancestral mtDNA sequence of the two *P. naumanni* specimens was reconstructed, and the divergence time of *P. naumanni* among the other extinct species of the WE clade was estimated using these ancestral sequences.

The ancestral sequence was reconstructed using the ML method in the programs BASEML and CODEML of PAML ver. 4.9.^55^ In ML phylogenetic inference,^65^ the likelihood of the observed sequences is calculated by summing over all possible ancestral states at all internal nodes. Based on the resulting ML tree and model parameters, the most probable nucleotide state can then be inferred for each ancestral node. We applied this principle to reconstruct ancestral sequences. Under the assumption of the tree topology of the ML tree as shown in Figure S6, sequences of all internal nodes including that of the MRCA of the two *P. naumanni* individuals, which was used in downstream analyses, were reconstructed. The ancestral sequences of the RNAs and D-loop were reconstructed under the GTR+Γ model, and those of the concatenated 12 protein-coding genes and *ND6* were reconstructed under the codon substitution+Γ model.^66^ If either of the two *P. naumanni* individuals had missing data at a nucleotide site, that site was also treated as missing in the reconstructed ancestral sequences. In the final alignment, 13,525 bp of the inferred ancestral sequence was used for analysis. Divergence times were estimated using BEAST ver. 1.10.4.^56^ The aforementioned five partitions used for inferring the ML tree were also applied, and the HKY+I+Γ model was applied for each partition. The uncorrelated relaxed clock model with the log-normal relaxed distribution was applied under the coalescent model with the assumption of a constant population size. The Markov chain Monte Carlo method was conducted for 100,000,000 generations, and trees were sampled every 10,000 generations. The first 10% of trees were discarded as burn-in.

The tip-dating method was applied for estimating divergence times. The age of the *P. antiquus* specimens from Neumark-Nord is 0.092–0.142 Ma, that of *P. antiquus* from Sicily is 0.05–0.175 Ma, that of the elephantid from Weimar-Ehringsdorf is 0.216–0.25 Ma, and that of the elephantid from Yangyuan is > 0.0503 Ma. Normal distributions were applied for prior distributions of these tip dates. Mean values for the tips were assumed to be the average of the lower and upper limits of each fossil date (with the exception that the mean of Yangyuan elephantid fossil was assumed to be 0.0503), and each corresponding standard deviation was assumed to be 0.05 Ma. The time of the most recent common ancestor (tMRCA) of the two *P. naumanni* specimens was assumed to be older than 0.049880 Ma (the ^14^C date of the fossil used in this study) and younger than 0.43 Ma, which is the estimated time of the land bridge connecting the Japanese Archipelago to the East Asian continent, thereby creating a potential path for *P. naumanni* hypothesised migration into Japan. A normal distribution was assumed for the tMRCA of *P. naumanni*, with a mean of 0.2 Ma and standard deviation of 0.1 Ma. We further applied a lognormal prior with a mean of 7.5 million years and standard deviation of 1 million years for the divergence of the *Loxodonta* and *Elephas* lineages.^67^

#### Validation of divergence-time estimates by ancestral-node reconstruction, read down-sampling, and error simulation

Ancient mtDNA can inflate terminal branches through postmortem damage and PCR duplicates, which may bias time inference that relies on terminal branch lengths. To mitigate such effects, we evaluated a procedure that first reconstructs ancestral sequences at internal nodes and then estimates divergence times from these reconstructions. Conceptually, this parallels Bayesian dating in which branch lengths and ancestral states are estimated jointly, while effectively allowing terminal branches to vary within bounds set by node ages. As independent test data, we used UDG-treated *P. antiquus* mitogenomes from Neumark-Nord (NEPEC, NEU2A), keeping the comparison aligned with the two *P. naumanni* sequences analyzed in this study (the third Neumark-Nord individual, NEU8B, was excluded for comparability). Reads were dereplicated and clustered at 93% similarity with VSEARCH to reduce PCR-derived duplicates. For our *P. naumanni* dataset, mapping after clustering still showed uneven coverage despite nominal coverage >8× (Figure S5), consistent with over-amplification of a few template molecules. In the Neumark-Nord *P. antiquus* data, maximum-likelihood trees inferred from down-sampled datasets likewise exhibited unusually long terminal branches (Figure S8).

To evaluate robustness under reduced effective coverage, we prepared inferred ancestral-sequence datasets at five levels: no down-sampling and down-sampling to 1,000, 500, 300, and 100 reads (Figure S9). The full datasets contained ∼5,000 reads for NEU2A and ∼15,000 for NEPEC. For dating, we used the same BEAST settings as in the primary analysis (five partitions, HKY+I+Γ for each, an uncorrelated log-normal relaxed clock, and a constant-size coalescent prior; see Estimating divergence times among *P. naumanni*). Posterior estimates of the divergence time between the NN ancestral node and the Sicilian individual (i.e., the MRCA uniting the NN clade and the Sicilian lineage) were then compared across down-sampling conditions (Figures S10 and S11).

To examine sensitivity to elevated error despite UDG treatment, we introduced 1% random substitutions into both full-length and down-sampled consensus sequences and into raw reads prior to consensus calling, then repeated ancestral-node reconstruction and dating (Figures S12 and S13). The 1% level was chosen as a conservative bound given *P. naumanni* terminal branch lengths of 0.003–0.004 (≈0.3–0.4% if entirely due to postmortem change; Figure S6). Across these analyses, posterior estimates remained concordant with complete-sequence results and centered around ∼0.4 Ma down to ∼300 reads, whereas noticeable fluctuations emerged at the 100-read condition.

### Quantification and statistical analysis

Phylogenetic relationships were inferred using maximum likelihood (ML) methods, and node support was assessed with 1,000 ultrafast bootstrap replicates implemented in IQ-TREE v2.1.3. To evaluate alternative topologies for the phylogenetic placement of *Palaeoloxodon naumanni*, we conducted Kishino–Hasegawa (KH) and Shimodaira–Hasegawa (SH) tests. Divergence time estimation was performed in BEAST v1.10.4 under an uncorrelated log-normal relaxed clock and a constant-size coalescent prior. Tip dating was based on the radiocarbon ages of fossil specimens from which DNA sequences were obtained, and the root age of the tree was constrained according to divergence estimates calibrated across Proboscidean evolution. We report median node ages with 95% highest posterior density (HPD) intervals. No data points were excluded.

## References

[1] Maglio V.J. (1973). Origin and evolution of the Elephantidae. Trans. Am. Philos. Soc..

[2] Shoshani J., Golenberg E.M., Yang H. (1998). Elephantidae phylogeny: morphological versus molecular results. Acta Theriol..

[3] Markov G., Saegusa H. (2008). On the validity of *Stegoloxodon* Kretzoi, 1950 (Mammalia: Proboscidea). Zootaxa.

[4] Sanders W.J., Gheerbrant E., Harris J.M., Saegusa H., Delmer C., Werdelin L., Sanders W.J. (2010). Cenozoic Mammals of Africa.

[5] Lister A.M. (2016). Dating the arrival of straight-tusked elephant (*Palaeoloxodon* spp.) in Eurasia. Bull. Mus. Anthropol. Préhist. Monaco Suppl..

[6] Larramendi A., Zhang H., Palombo M.R., Ferretti M.P. (2020). The evolution of *Palaeoloxodon* skull structure: disentangling phylogenetic, sexually dimorphic, ontogenetic, and allometric morphological signals. Quat. Sci. Rev..

[7] Matsumoto H. (1924). Preliminary notes on fossil elephants in Japan. J. Geol. Soc. Jpn..

[8] Beden M., Harris J.M. (1983). Koobi Fora Research Project, the Fossil Ungulates: Proboscidea, Perissodactyla, and Suidae.

[9] Saegusa H., Gilbert W.H., Gilbert W.H., Asfaw B. (2008). *Homo erectus* in Africa: Pleistocene evidence from the Middle Awash.

[10] Shoshani J., Tassy P. (1996).

[11] Meyer M., Palkopoulou E., Baleka S., Stiller M., Penkman K.E.H., Alt K.W., Ishida Y., Mania D., Mallick S., Meijer T. (2017). Palaeogenomes of Eurasian straight-tusked elephants challenge the current view of elephant evolution. eLife.

[12] Palkopoulou E., Lipson M., Mallick S., Nielsen S., Rohland N., Baleka S., Karpinski E., Ivancevic A.M., To T.H., Kortschak R.D. (2018). A comprehensive genomic history of extinct and living elephants. Proc. Natl. Acad. Sci. USA.

[13] Todd N.E. (2010). New phylogenetic analysis of the family Elephantidae based on cranial-dental morphology. Anat. Rec..

[14] Falconer H., Cautley P.T. (1845). the North of India.

[15] Todd N.E. (2010). Qualitative comparison of the cranio-dental osteology of the extant elephants, *Elephas maximus* (Asian Elephant) and *Loxodonta africana* (African Elephant). Anat. Rec..

[16] Lin H., Hu J., Baleka S., Yuan J., Chen X., Xiao B., Song S., Du Z., Lai X., Hofreiter M., Sheng G. (2023). A genetic glimpse of the Chinese straight-tusked elephants. Biol. Lett..

[17] Matschie P. (1900). Über geographische Abarten des afrikanischen Elefanten. Sitzungsberichte Ges. Naturforschender Freunde Berl..

[18] Makiyama J. (1924). Notes on a fossil elephant from Sahamma. Tôtômi. Mem. Coll. Sci. Kyoto Imp. Univ. Ser. B.

[19] Inuzuka N. (1977). On the origin of *Palaeoloxodon naumanni* –A comparative osteology of the cranium. J. Geol. Soc. Jpn..

[20] Inuzuka N. (1977). Measurements of tusks of *Palaeoloxodon naumanni*. Earth Sci..

[21] Takahashi K. (2022). An overview of *Palaeloxodon naumanni*, the *Palaeoloxodon* (Elephantidae) of the far east: distribution, morphology and habitat. Hist. Biol..

[22] Nihonbashi Naumann’s Elephant Research Group (1981). Fossil bones of the Naumann’s elephant, *Palaeoloxodon naumanni*, from Nihonbashi Hama-cho, Chuo-ku, Tokyo. Tokyo-to Maizou Bunkazai Chousa Houkoku [Tokyo Archaeological Records].

[23] Hasegawa Y. (1972). The Naumann’s Elephant, *Palaeoloxodon naumanni* (Makiyama) from the Late Pleistocene off Shakagahana, Shodoshima Is. In Seto Inland Sea, Japan. Bull. Nat. Sci. Mus..

[24] Matsu'ura K., Kondo M., Prefecture I., Dodo Y., Takigawa W., Sawada J. (2003). Search for Japanese Pleistocene Human Fossils in the Kitakami Mountains-Excavation of Abakuchi and Kazaana Cave sites in Osako Town.

[25] Falconer H., Cautley P.T. (1847). the North of India.

[26] Adams A.L. (1874). On the dentition and osteology of the Maltese fossil elephant, being a description of the remains discovered by the author in Malta between the years 1860 and 1866. Trans. Zool. Soc. Lond..

[27] Dubrovo I.A. (1960). The ancient elephant in USSR (in Russian). Tr. Paleontol. Inst. Akad. Nauk SSSR.

[28] Liu J. (1977). *Palaeoloxodon* from Huaiyuan District, northern part of Anhui. Vertebr. Palasiat..

[29] Larramendi A., Palombo M.R., Marano F. (2017). Reconstructing the life appearance of a Pleistocene giant: size, shape, sexual dimorphism and ontogeny of *Palaeoloxodon antiquus* (Proboscidea: Elephantidae) from Neumark-Nord 1 (Germany). Boll. Soc. Paleontol. Ital..

[30] Tada R. (1997). Paleoenvironmental changes in and around the Japan Sea since the Last Glacial Period. Quat. Res. (Daiyonki-Kenkyu).

[31] Ishida Y., Georgiadis N.J., Hondo T., Roca A.L. (2013). Triangulating the provenance of African elephants using mitochondrial DNA. Evol. Appl..

[32] Zhang H. (2020).

[33] Sanders W.J. (2023).

[34] Sonakia A., Biswas S. (2011). Fossil mammals including early man from the Quaternary deposits of the Narmada and Son basins of Madhya Pradesh, India. Palaeontol. Indica.

[35] Turvey S.T., Sathe V., Crees J.J., Jukar A.M., Chakraborty P., Lister A.M. (2021). Late Quaternary megafaunal extinctions in India: How much do we know?. Quat. Sci. Rev..

[36] Sankhyan A.R. (2020). Evolutionary perspective on Narmada hominin fossils. Adv. Anthropol..

[37] Patnaik R., Singh N.P., Paul D., Sukumar R. (2019). Dietary and habitat shifts in relation to climate of Neogene-Quaternary proboscideans and associated mammals of the Indian subcontinent. Quat. Sci. Rev..

[38] Athanassiou A., Vlachos E. (2022). Fossil Vertebrates of Greece, Vol. 1: Basal vertebrates, Amphibians, Reptiles, Afrotherians, Glires, and Primates.

[39] Mualim K., Theunert C., Slatkin M. (2021). Estimation of coalescence probabilities and population divergence times from SNP data. Heredity.

[40] Jukar A.M., Bhat G.M., Parfitt S., Ashton N., Dickinson M., Zhang H., Dar A.M., Lone M.S., Thusu B., Craig J. (2024). A remarkable *Palaeoloxodon* (Mammalia, Proboscidea) skull from the intermontane Kashmir Valley, India. J. Vertebr. Paleontol..

[41] Segawa T., Yonezawa T., Mori H., Akiyoshi A., Allentoft M.E., Kohno A., Tokanai F., Willerslev E., Kohno N., Nishihara H. (2021). Ancient DNA reveals multiple origins and migration waves of extinct Japanese brown bear lineages. R. Soc. Open Sci..

[42] Segawa T., Yonezawa T., Mori H., Kohno A., Kudo Y., Akiyoshi A., Wu J., Tokanai F., Sakamoto M., Kohno N., Nishihara H. (2022). Paleogenomics reveals independent and hybrid origins of two morphologically distinct wolf lineages endemic to Japan. Curr. Biol..

[43] Waku D., Segawa T., Yonezawa T., Akiyoshi A., Ishige T., Ueda M., Ogawa H., Sasaki H., Ando M., Kohno N., Sasaki T. (2016). Evaluating the phylogenetic status of the extinct Japanese otter on the basis of mitochondrial genome analysis. PLoS One.

[44] Wu J., Kohno N., Mano S., Fukumoto Y., Tanabe H., Hasegawa M., Yonezawa T. (2015). Phylogeographic and demographic analysis of the Asian black bear (*Ursus thibetanus*) based on mitochondrial DNA. PLoS One.

[45] Takahashi K., Namatsu K. (2000). Origin of the Japanese Proboscidea in the Plio-Pleistocene. Earth Sci..

[46] Wei Q. (1976). Recent find of fossil *Palaeoloxodon namadicus* from Nihewan Beds, NW Hebei. Vertebr. Palasiat..

[47] Chen S., Zhou Y., Chen Y., Gu J. (2018). fastp: an ultra-fast all-in-one FASTQ preprocessor. Bioinformatics.

[48] Li H., Durbin R. (2009). Fast and accurate short read alignment with Burrows-Wheeler transform. Bioinformatics.

[49] Rognes T., Flouri T., Nichols B., Quince C., Mahé F. (2016). VSEARCH: a versatile open source tool for metagenomics. PeerJ.

[50] Thorvaldsdóttir H., Robinson J.T., Mesirov J.P. (2012). Integrative Genomics Viewer (IGV): high-performance genomics data visualization and exploration. Brief Bioinform.

[51] Jónsson H., Ginolhac A., Schubert M., Johnson P.L.F., Orlando L. (2013). mapDamage2.0: fast approximate Bayesian estimates of ancient DNA damage parameters. Bioinformatics.

[52] Dolenz S., van der Valk T., Jin C., Oppenheimer J., Sharif M.B., Orlando L., Shapiro B., Dalén L., Heintzman P.D. (2024). Unravelling reference bias in ancient DNA datasets. Bioinformatics.

[53] Katoh K., Standley D.M. (2013). MAFFT multiple sequence alignment software version 7: improvements in performance and usability. Mol. Biol. Evol..

[54] Nguyen L.T., Schmidt H.A., von Haeseler A., Minh B.Q. (2015). IQ-TREE: a fast and effective stochastic algorithm for estimating maximum-likelihood phylogenies. Mol. Biol. Evol..

[55] Yang Z. (2007). PAML 4: phylogenetic analysis by maximum likelihood. Mol. Biol. Evol..

[56] Suchard M.A., Lemey P., Baele G., Ayres D.L., Drummond A.J., Rambaut A. (2018). Bayesian phylogenetic and phylodynamic data integration using BEAST 1.10. Virus Evol..

[57] Hasegawa Y., Sotsuka T., Urata K. (1980). Preliminary report on two fossil baby elephant, *Palaeoloxodon naumanni* from Seiryukutsu-cave deposits on Hiraodai karst plateau, northern Kyushu, Japan. Bull. Kitakyushu Mus. Nat. Hist..

[58] Inuzuka N., Sawamura H. (1985). Sexuality in the cranial form of *Palaeoloxodon naumanni*. J. Fossil Res..

[59] Takahashi K., Shimaguchi T., Kamiya H. (2006). *Palaeoloxodon naumanni* fossils from Shikkari, Higashi-doori, Shimokita-gun, Aomori Prefecture, Japan and its AMS-14C dating. J. Fossil Res..

[60] Hasegawa Y., Tomida Y., Kohno N., Ono K., Nokariya H., Uyeno T. (1988). Quaternary vertebrates from Shiriya area, Shimokita Peninsula, northern Japan. Mem. Natl. Sci. Mus..

[61] Shimaguchi T. (2001). Fossil elephant molars in the Aomori Prefectural Museum. Bull. Aomori Prefect. Mus..

[62] Orlando L., Ginolhac A., Zhang G., Froese D., Albrechtsen A., Stiller M., Schubert M., Cappellini E., Petersen B., Moltke I. (2013). Recalibrating *Equus* evolution using the genome sequence of an early Middle Pleistocene horse. Nature.

[63] Rohland N., Glocke I., Aximu-Petri A., Meyer M. (2018). Extraction of highly degraded DNA from ancient bones, teeth and sediments for high-throughput sequencing. Nat. Protoc..

[64] Gansauge M.T., Aximu-Petri A., Nagel S., Meyer M. (2020). Manual and automated preparation of single-stranded DNA libraries for the sequencing of DNA from ancient biological remains and other sources of highly degraded DNA. Nat. Protoc..

[65] Felsenstein J. (1981). Evolutionary trees from DNA sequences: a maximum likelihood approach. J. Mol. Evol..

[66] Yang Z., Nielsen R., Hasegawa M. (1998). Models of amino acid substitution and applications to mitochondrial protein evolution. Mol. Biol. Evol..

[67] Rohland N., Malaspinas A.S., Pollack J.L., Slatkin M., Matheus P., Hofreiter M. (2007). Proboscidean mitogenomics: chronology and mode of elephant evolution using mastodon as outgroup. PLoS Biol..

