# Supplementary material for: Ancient DNA from Palaeoloxodon naumanni in Japan reveals early evolution of Eurasian Palaeoloxodon
Source: iScience. 2025 Nov 21;28(12):114156. doi: 10.1016/j.isci.2025.114156 (PMC12741397; doi:10.1016/j.isci.2025.114156)
Supplement: Document S1. Figures S1–S14 and Tables S1 and S2 [file mmc1.pdf]

iScience, Volume *28*

Supplemental information

Ancient DNA from *Palaeoloxodon naumanni* in Japan reveals early evolution of Eurasian *Palaeoloxodon*

Takahiro Segawa, Takahiro Yonezawa, Hiroshi Mori, Ayumi Akiyoshi, Asier Larramendi, and Naoki Kohno

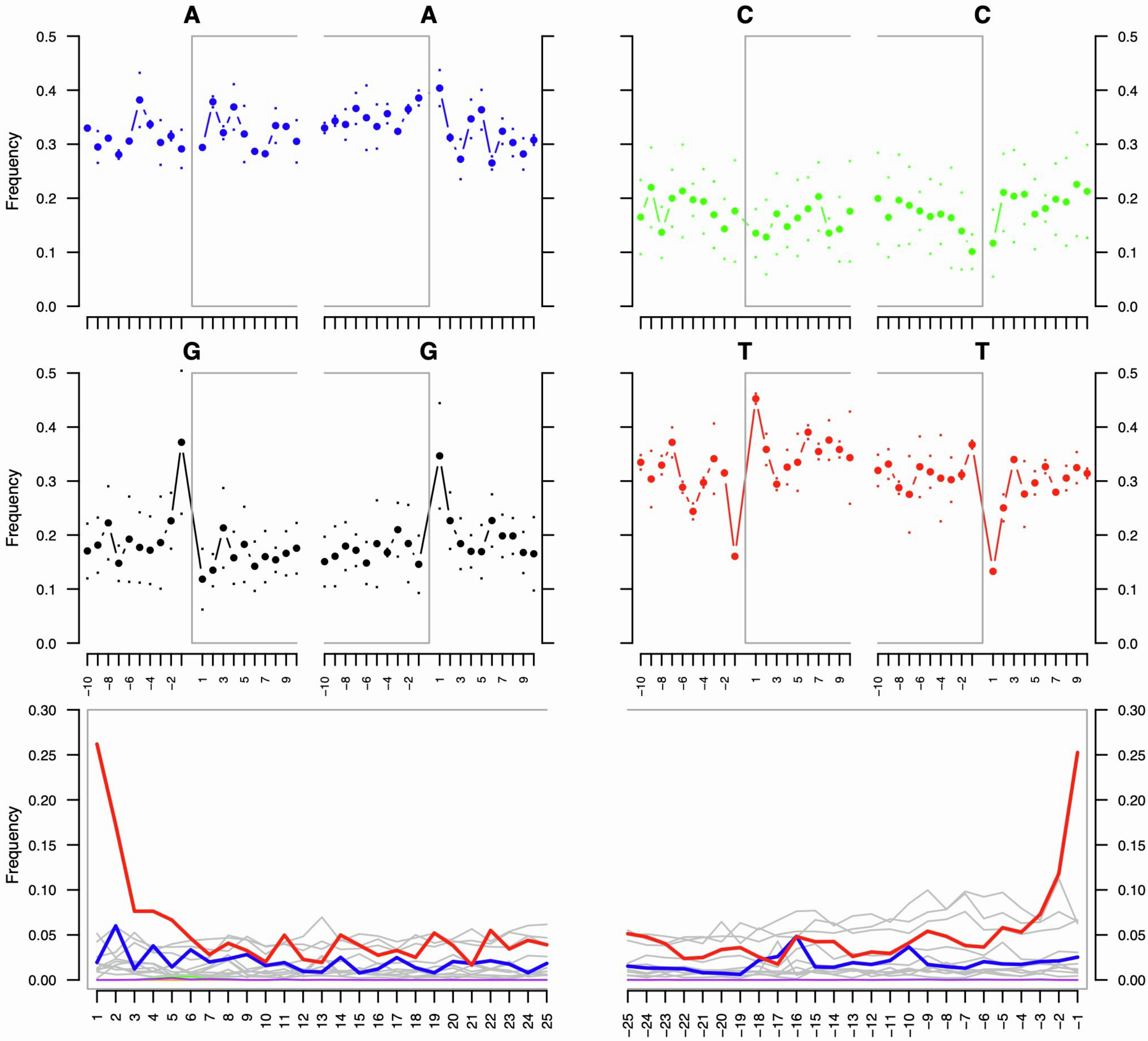

Figure S1. Ancient DNA signal analysis using MapDamage for NMNS-PV 22670.
Characteristics of postmortem DNA damage patterns were observed in the DNA sample of NMNS-PV 22670 using MapDamage. The x-axis represents the relative nucleotide positions along the DNA reads from both ends. The red line indicates the frequency of C→T substitutions, while the blue line indicates G→A substitutions. The notable increase in C→T substitutions near the ends of the reads is a typical indicator of ancient DNA damage.

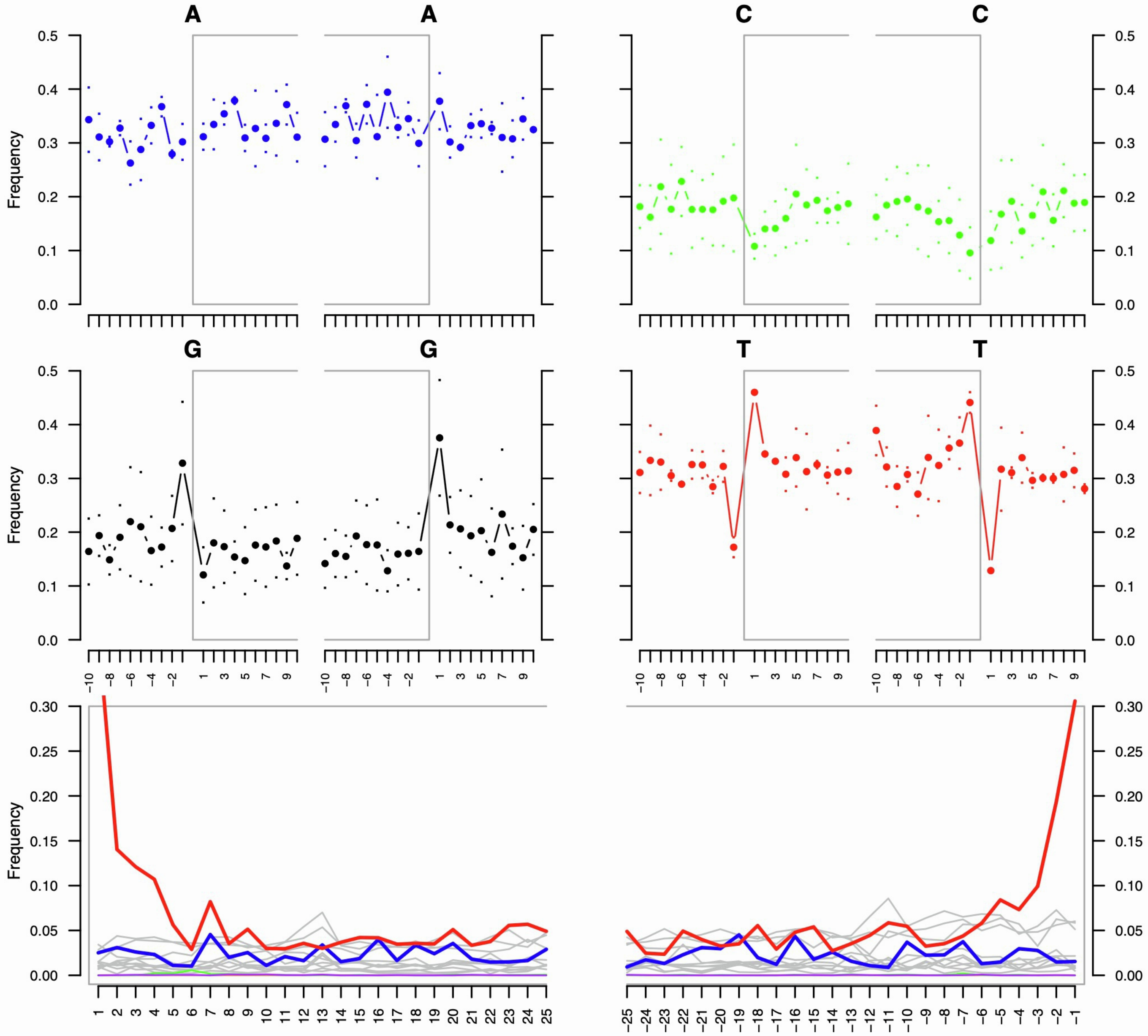

Figure S2. Ancient DNA signal analysis using MapDamage for AoPM 1975-7.

Characteristics of postmortem DNA damage patterns were observed in the DNA sample of AoPM 1975-7 using MapDamage. The x-axis represents the relative nucleotide positions along the DNA reads from both ends. The red line indicates the frequency of C→T substitutions, while the blue line indicates G→A substitutions. The notable increase in C→T substitutions near the ends of the reads is a typical indicator of ancient DNA damage.

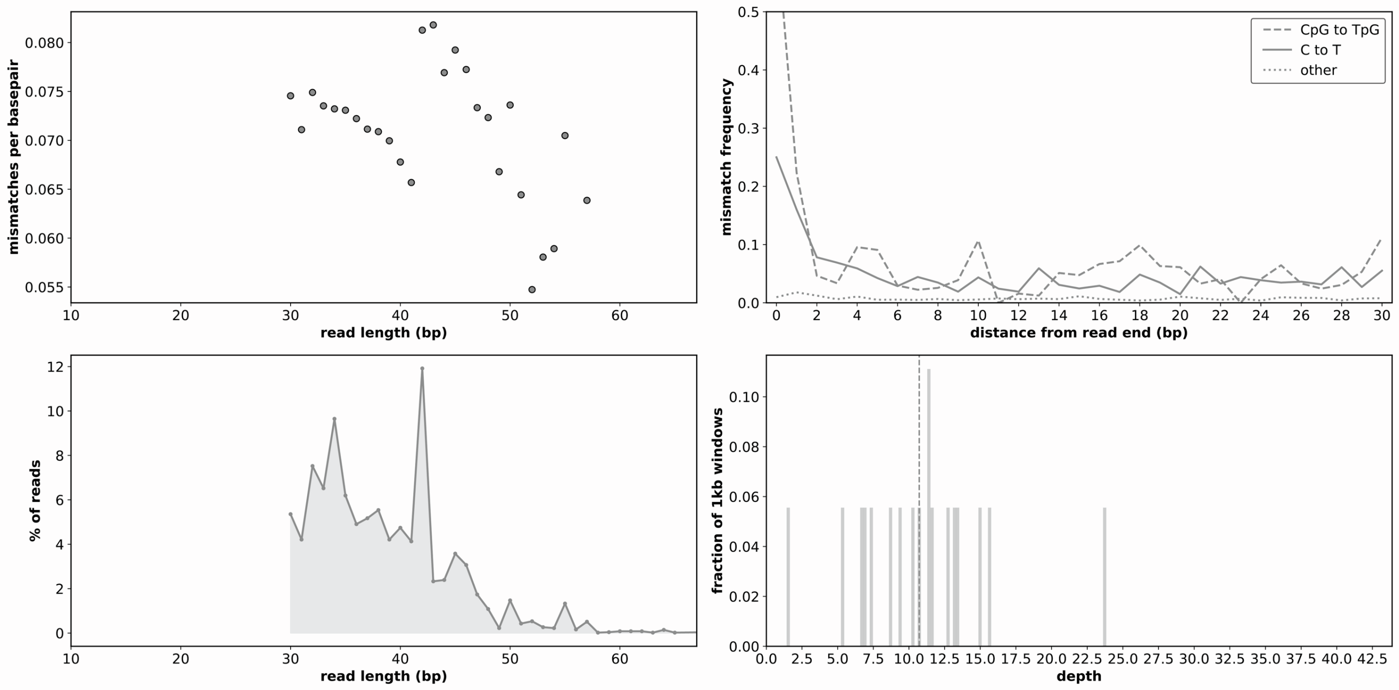

Figure S3. AMBER analysis result of NMNS-PV 22670. The ancient DNA mapping quality assessment tool AMBER was applied to the NMNS-PV 22670 mitochondrial read mapping result against the reference sequence (AB443879.1). Read length vs. % of mismatches per base pair (top left), distance from read end vs. mismatch frequencies of CpG to TpG and C to T (top right), read length vs. % of reads (bottom left), and read depth vs. fraction of 1kb windows along the reference sequence (bottom right) are plotted.

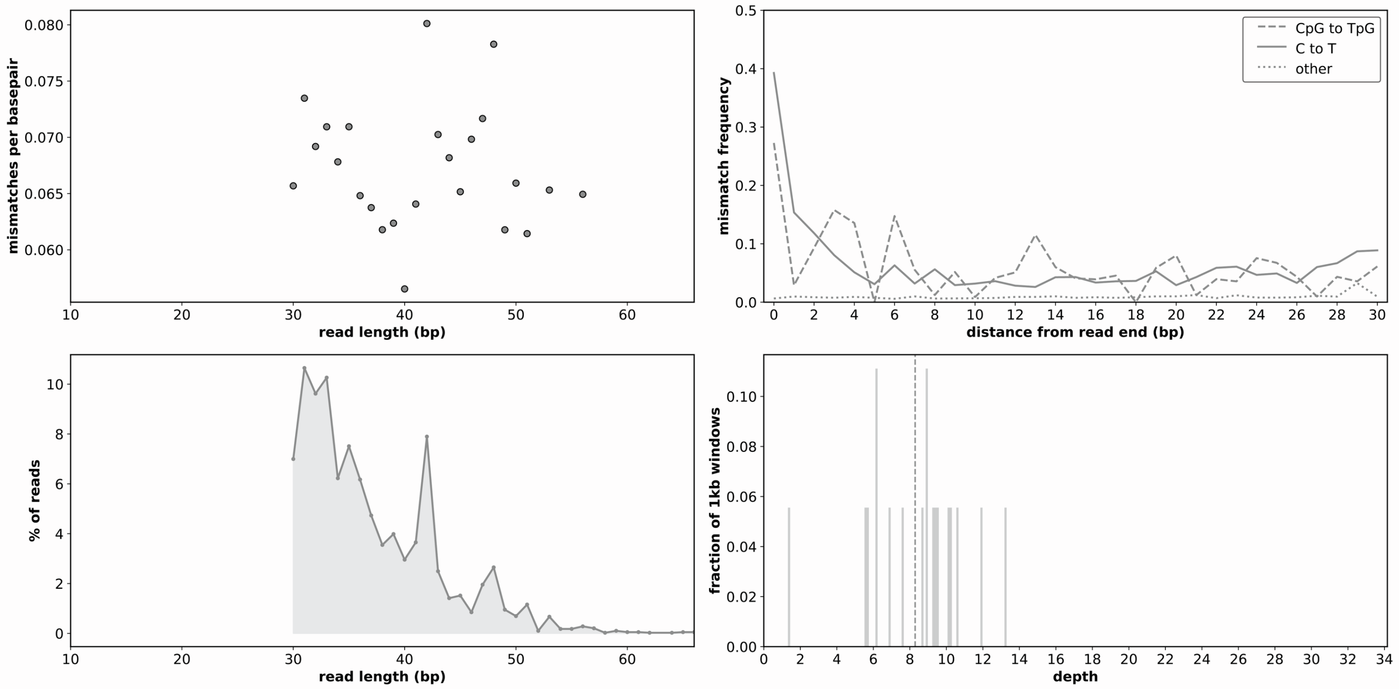

Figure S4. AMBER analysis result of AoPM 1975-7. The ancient DNA mapping quality assessment tool AMBER was applied to the AoPM 1975-7 mitochondrial read mapping result against the reference sequence (AB443879.1). Read length vs. % of mismatches per base pair (top left), distance from read end vs. mismatch frequencies of CpG to TpG and C to T (top right), read length vs. % of reads (bottom left), and read depth vs. fraction of 1kb windows along the reference sequence (bottom right) are plotted.

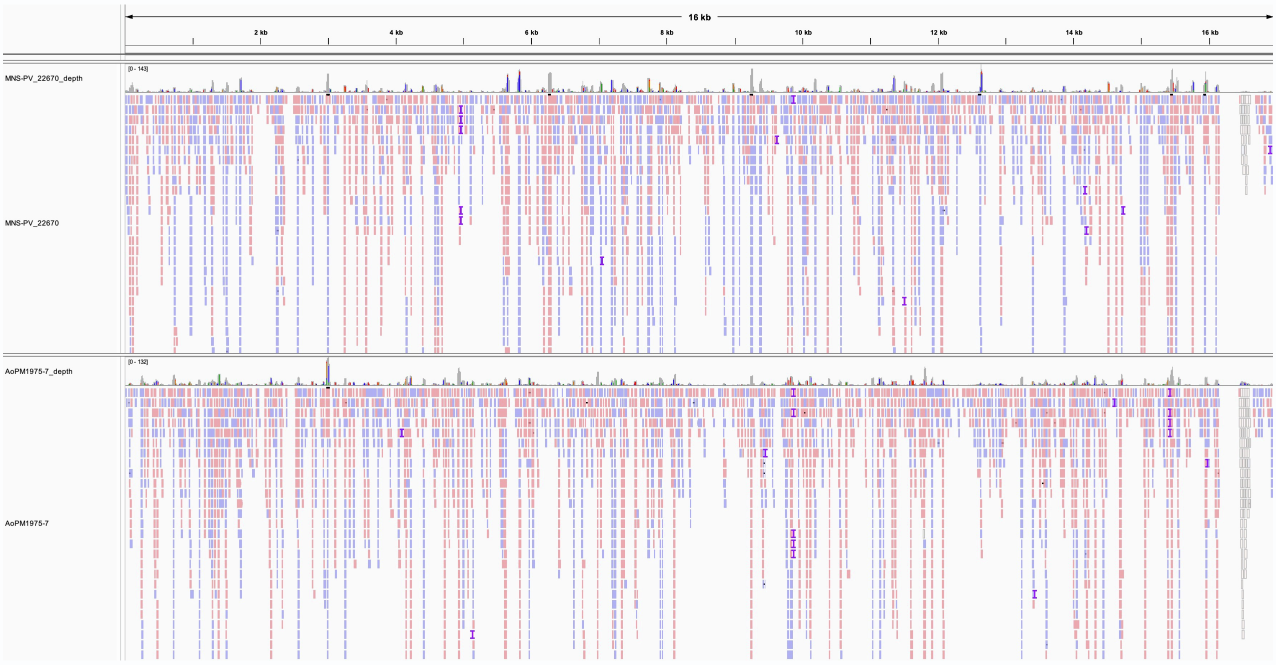

Figure S5. Read distributions for samples NMNS-PV 22670 and AoPM 1975-7. Mapped reads against the reference mitochondrial genome sequence (AB443879.1) are visualized in IGV. Reads colored blue and red indicate mapping to the plus and minus strands, respectively. Regions of high read depth are primarily attributable to PCR duplicates. Consensus sequences were generated using only positions covered by at least 70 independent reads. The reconstructed mitochondrial genomes cover over 90% of the reference sequence, with coverage breadths of 93.23% for NMNS-PV 22670 and 94.71% for AoPM 1975-7, respectively.

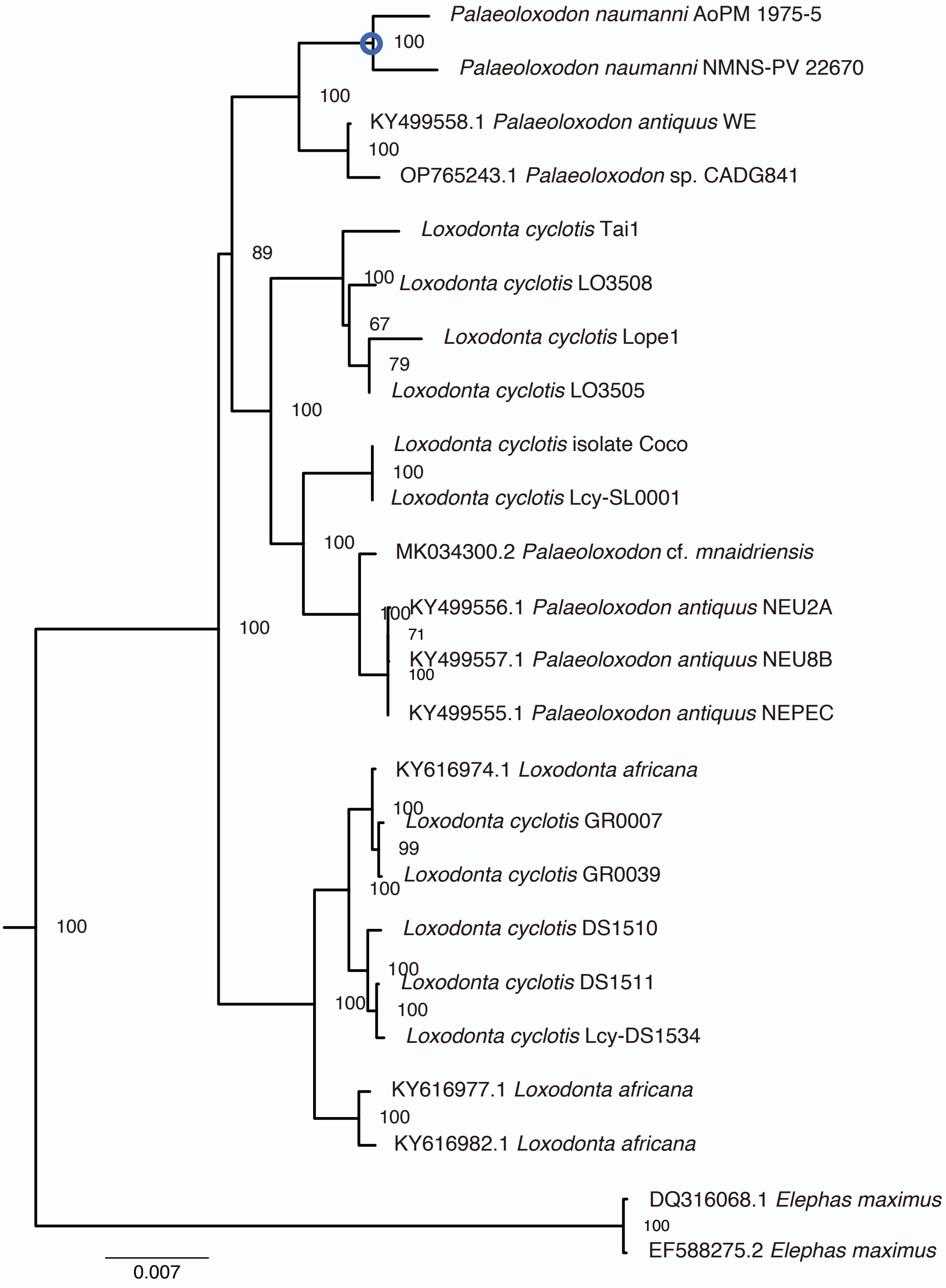

Figure S6. Maximum likelihood (ML) tree as inferred from the complete mitochondrial genomes of the Elephantidae. The branch lengths are proportional to the numbers of the nucleotide substitution. The nodal numbers indicate the ultrafast bootstrap probabilities with 1000 replications. The node of the most recent common ancestor of two *P. naumanni* (NMNS-PV 22670 and AoPM 1975-7) was marked by the blue colored open circle. The nucleotide sequence of this ancestral node was reconstructed by the ML method and used for the Bayesian inference of the time tree shown in Figure 1.

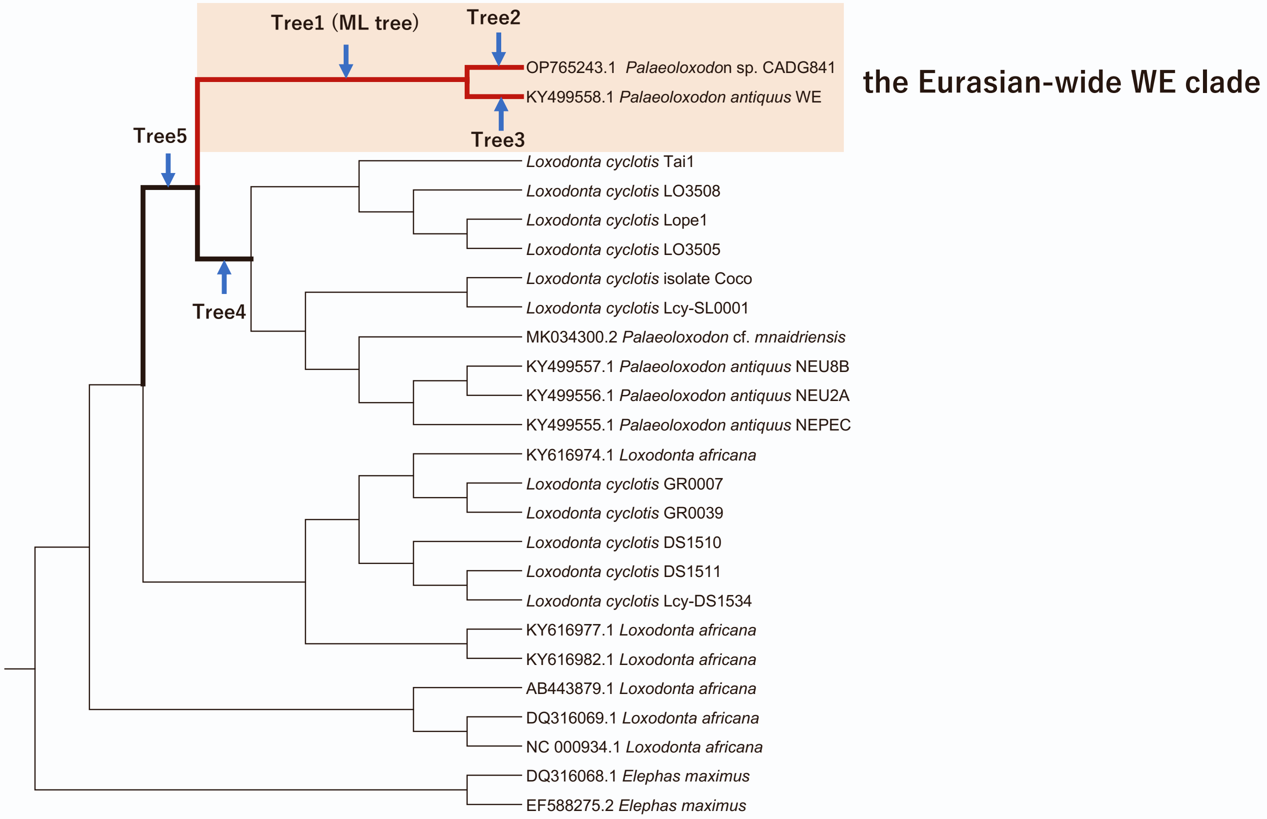

Figure S7. Phylogenetic placement of *P. naumanni* within and around the Eurasian-wide WE clade. The phylogenetic positions of *P. naumanni* were evaluated through detailed statistical analyses. Clades or individual samples of *P. naumanni* were sequentially placed (blue arrows) onto five branches either within the Eurasian-wide WE clade (highlighted in dark red) or nearby (highlighted in black), and their likelihood scores were assessed using PAML. Tree 1 topology corresponds to the maximum likelihood tree obtained by a heuristic search using IQ-TREE 2 (Figure S6). Log-likelihood values for each topology, differences and standard deviations from the best-scoring tree, p-values from the Kishino-Hasegawa and Shimodaira-Hasegawa tests, and bootstrap values are provided in Table S2.

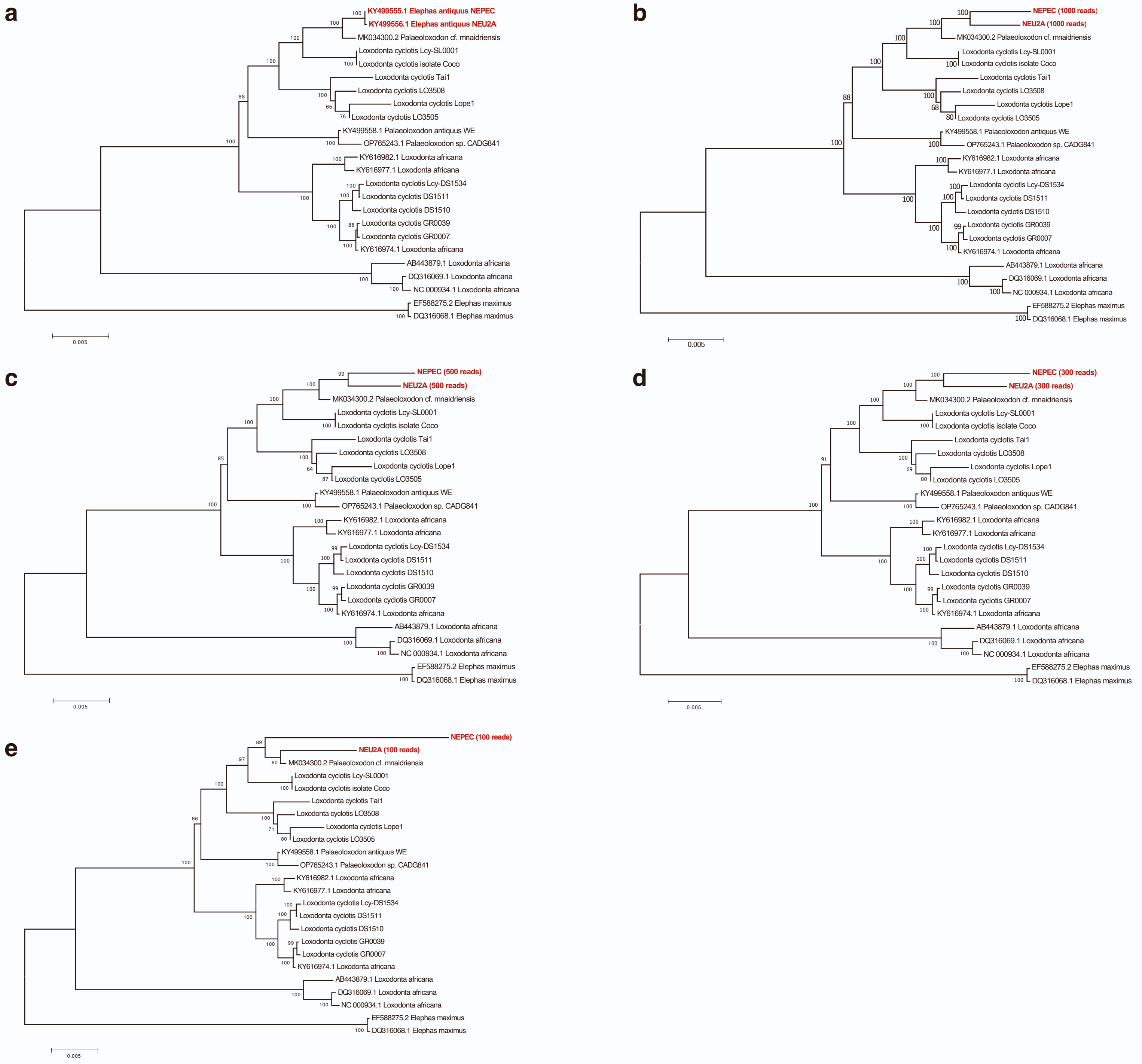

Figure S8. Effect of read down-sampling on phylogenetic tree estimation. For the mitochondrial genomes of *P. antiquus* individuals from Neumark-Nord (NEPEC and NEU2A), phylogenetic trees were inferred using the ML method with IQ-TREE 2, based on (**a**) complete sequences, (**b**) sequences constructed from data down-sampled to 1,000 reads, (**c**) sequences constructed from data down-sampled to 500 reads, (**d**) sequences constructed from data down-sampled to 300 reads, and (**e**) sequences constructed from data down-sampled to 100 reads. Down-sampling had minimal effect on the inferred tree topologies, all of which remained identical except for the topology inferred from the 100-read dataset. However, terminal branch lengths of the two Neumark-Nord individuals were notably affected by down-sampling. Data sets were separated into five partitions: concatenated 13 protein-coding genes (further separated into three codon positions, with ND6 using the complementary strand sequence), RNA gene (two rRNA and 22 tRNA, all converted to H-strand sequences), and the D-loop. The best fit substitution model for each partition was selected using the Bayesian Information Criterion (BIC). Branch lengths in the phylogenetic tree are proportional to the number of nucleotide substitutions. Nodal numbers indicate ultra-fast bootstrap probabilities (1,000 replications). The two Neumark-Nord individuals are highlighted in bold red font.

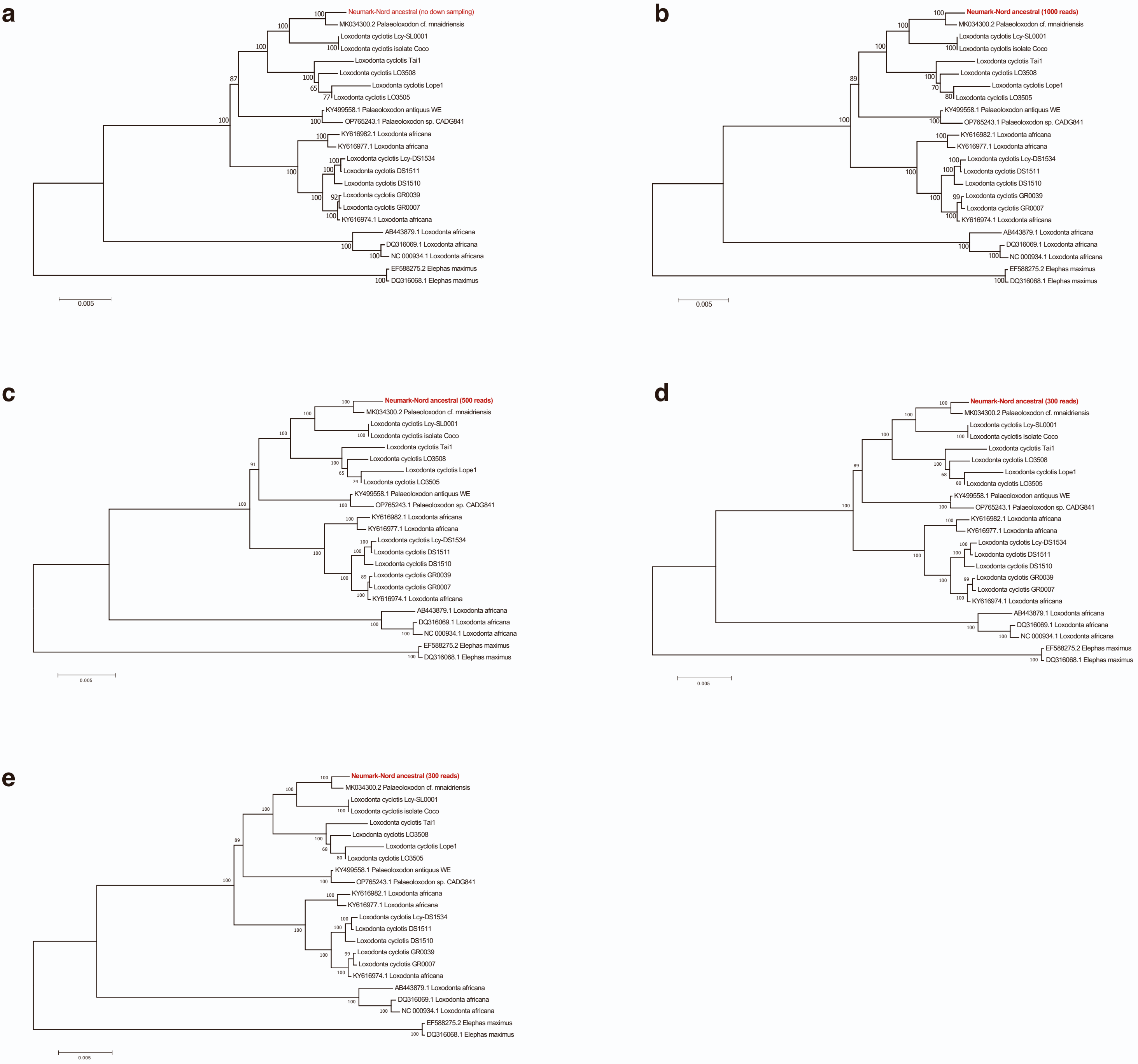

Figure S9. Effect of read down-sampling on ancestral sequence estimation. Ancestral mitochondrial genome sequences of two *P. antiquus* individuals from Neumark-Nord (NEPEC and NEU2A) were inferred under the following conditions: (**a**) without down-sampling, (**b**) down-sampled to 1,000 reads, (**c**) down-sampled to 500 reads, (**d**) down-sampled to 300 reads, and (**e**) down-sampled to 100 reads. Ancestral sequence estimation was performed using the CODEML program (for protein-coding genes) and the BASEML program (for RNA and D-loop) of PAML, based on the tree topology shown in Figure S8 (a-d). The CODEML analysis used a codon substitution model with a Γ distribution, separately analyzing the 12 protein-coding genes on the H-strand and ND6 to account for differences in base composition. The BASEML analysis used the GTR+Γ model. Bases missing in both Neumark-Nord individuals were treated as missing data in the ancestral sequence inference. The phylogenetic tree was estimated using IQ-TREE 2. Data sets were separated into five partitions: concatenated 13 protein-coding genes (further divided into three codon positions, with ND6 using the complementary strand sequence), RNA gene (two rRNA and 22 tRNA, all converted to H-strand sequences), and the D-loop. The best fit substitution model for each partition was selected using the BIC. Branch lengths in the phylogenetic tree represent the number of nucleotide substitutions, and nodal numbers indicate ultra-fast bootstrap probabilities (1,000 replications). The two Neumark-Nord individuals are highlighted in bold red font.

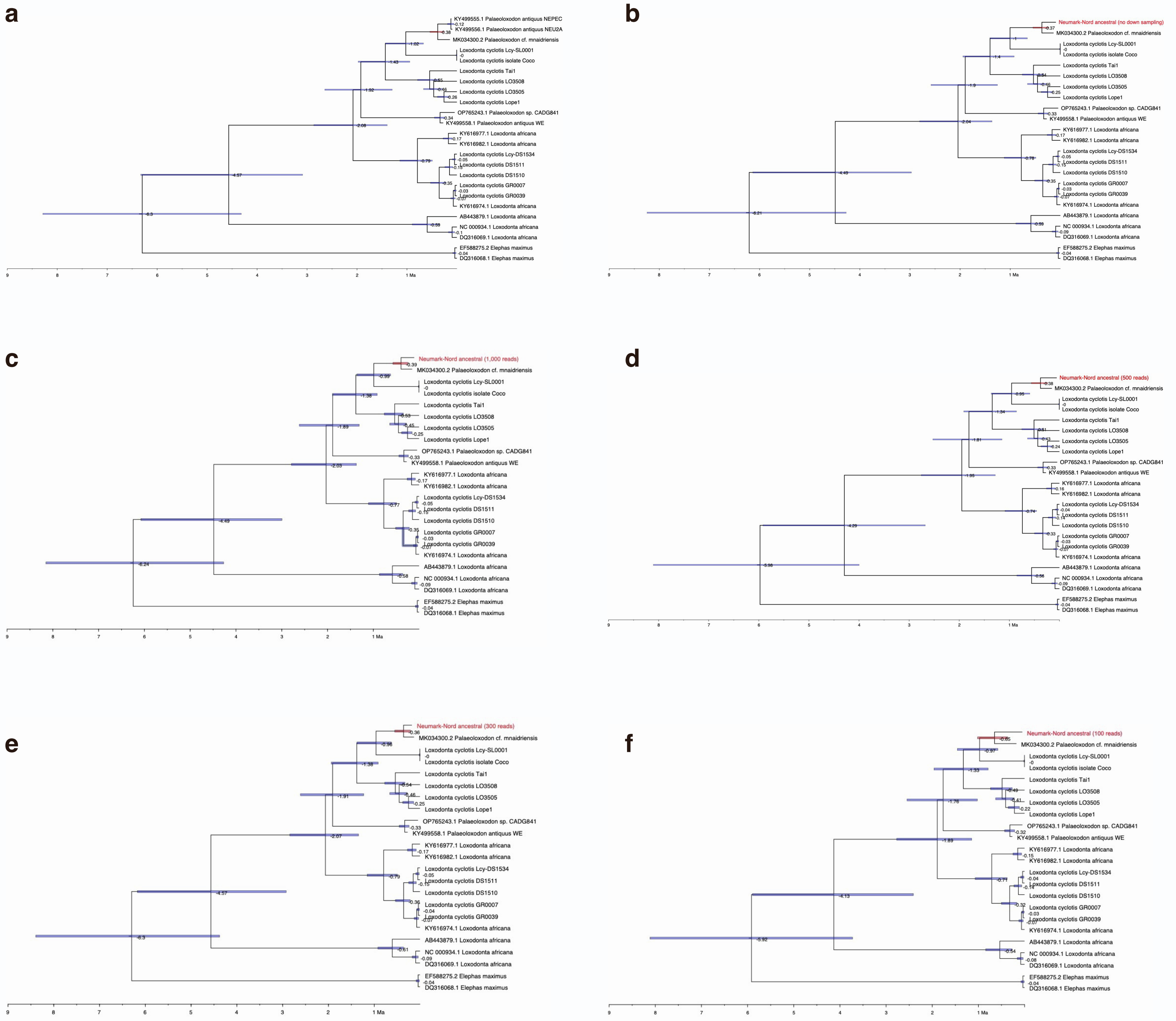

Figure S10. Effect of read down-sampling on time estimations. For the mitochondrial genomes of two *P. antiquus* individuals from Neumark-Nord (NEPEC and NEU2A), time estimations were performed using the following datasets: (**a**) complete sequence, (**b**) inferred ancestral sequence without down-sampling, (**c**) inferred ancestral sequence after down-sampling to 1,000 reads, (**d**) inferred ancestral sequence after down-sampling to 500 reads, (**e**) inferred ancestral sequence after down-sampling to 300 reads, and (**f**) inferred ancestral sequence after down-sampling to 100 reads. Coalescent times were estimated using BEAST, with data divided into five partitions: 13 protein-coding genes (further divided into three codon loci, using the complementary strand for ND6), RNA gene (two rRNA and 22 tRNA, converted to H-strand sequences), and the D-loop. The HKY+I+Γ model and a relaxed log-normal clock model were applied to each partition. Branch lengths in the phylogenetic tree represent estimated times, with nodal values indicating time estimates (Ma) and nodal bars showing their 95% highest posterior density (HPD). Nodal bars representing the tMRCA for the NN clade, which corresponds to the divergence time of the Sicilian and Neumark-Nord individuals, are highlighted in red. The two Neumark-Nord individuals are shown in bold red font. The X-axis represents time (Ma), and the Y-axis represents probability density. The posterior probability distributions based on the ancestral sequence estimated from the complete sequence closely align with those from the complete sequence itself. Down-sampling up to 300 reads has minimal effect on the posterior probability distribution, but significant fluctuations are observed when down-sampling to 100 reads.

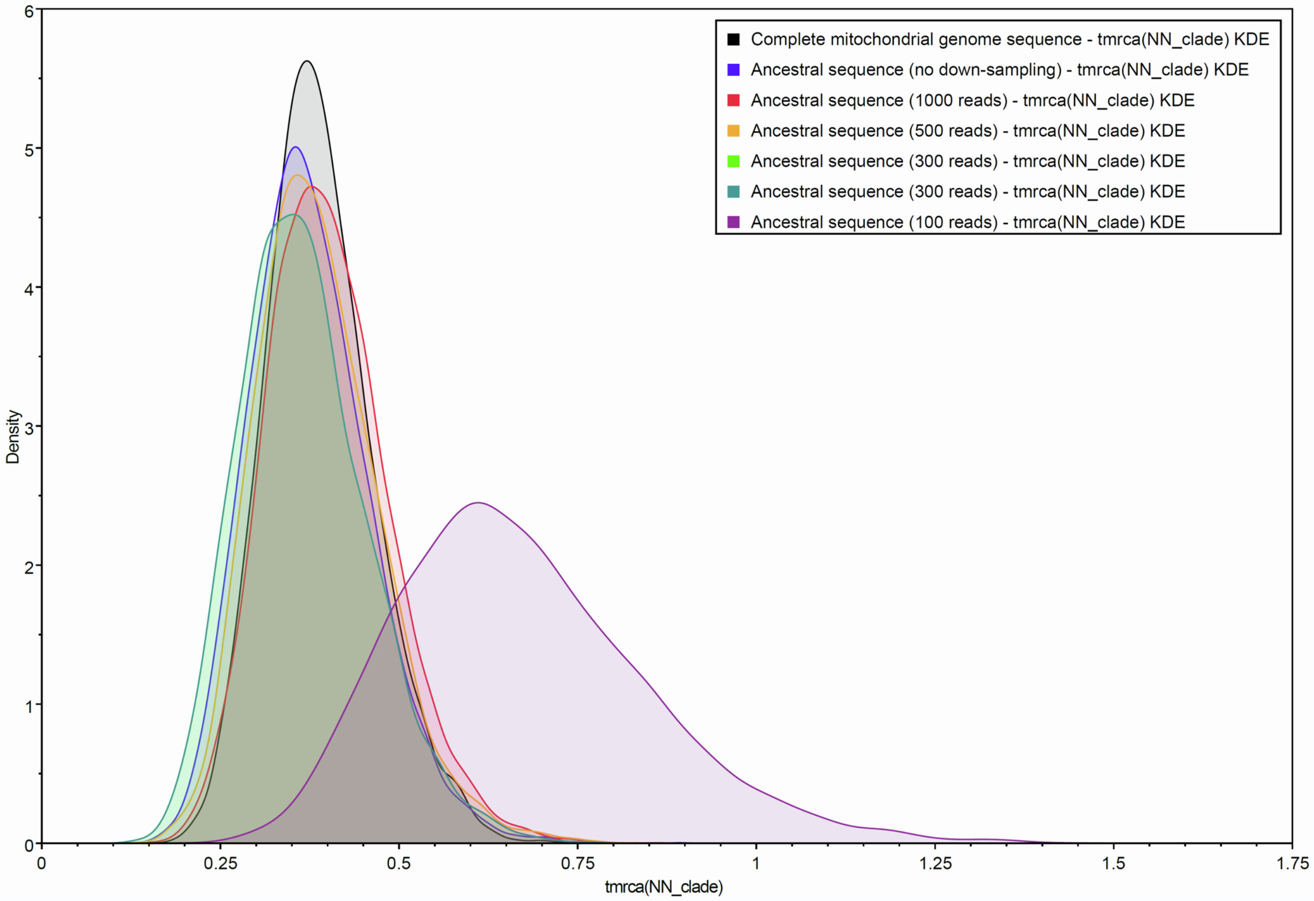

Posterior probability distribution of tMRCA for NN clades, estimated from both complete and down-sampled ancestral sequences.

Figure S11. Posterior probability distribution of the tMRCA for the NN clade.

The X-axis represents time (Ma), and the Y-axis represents probability density. The posterior probability distributions based on the ancestral sequence estimated from the complete sequence closely align with those from the complete sequence itself. Down-sampling up to 300 reads has minimal effect on the posterior probability distribution, but significant fluctuations are observed when down-sampling to 100 reads.

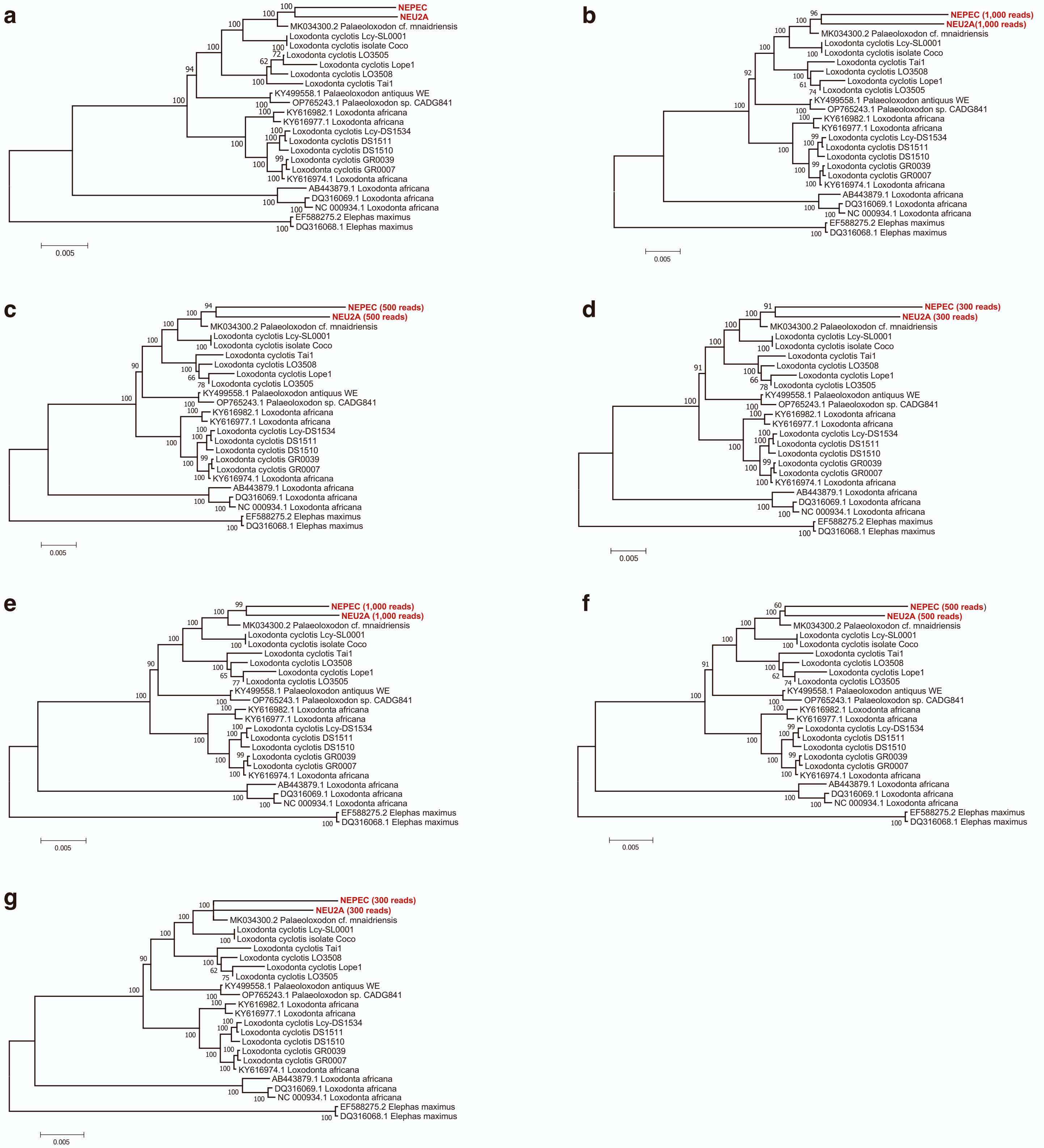

Figure S12. Effect of a high sequence error rate on phylogenetic tree estimation. Phylogenetic trees were inferred using the maximum likelihood method implemented in IQ-TREE 2, based on simulated mitochondrial genome sequences of two *P. antiquus* individuals from Neumark-Nord (NEPEC and NEU2A), with a 1% sequencing error artificially introduced. Errors were introduced either into (**a**–**d**) consensus sequences or (**e**–**g**) raw NGS reads prior to consensus sequence construction. Specifically: (**a**) errors introduced into complete consensus sequences; (**b**) errors introduced into consensus sequences built from 1,000 down-sampled reads; (**c**) 500 down-sampled reads; (**d**) 300 down-sampled reads; (**e**) error introduced into 1,000 down-sampled raw reads prior to consensus sequence construction; (**f**) 500 down-sampled raw reads; and (**g**) 300 down-sampled raw reads. The introduction of a high sequence error rate had minimal impact on tree topology inference, which remained identical across conditions. However, the terminal branch lengths of the two Neumark-Nord individuals were notably affected by the introduced errors. Sequences were separated into five partitions: concatenated 13 protein-coding genes (further separated by codon positions, with ND6 analyzed on the complementary strand), RNA genes (two rRNAs and 22 tRNAs, all converted to H-strand sequences), and the D-loop. The best-fit substitution model for each partition was selected based on the Bayesian Information Criterion (BIC). Branch lengths in the phylogenetic tree are proportional to the number of nucleotide substitutions. Nodal values represent ultrafast bootstrap support (1,000 replications). The two Neumark-Nord individuals are highlighted in bold red.

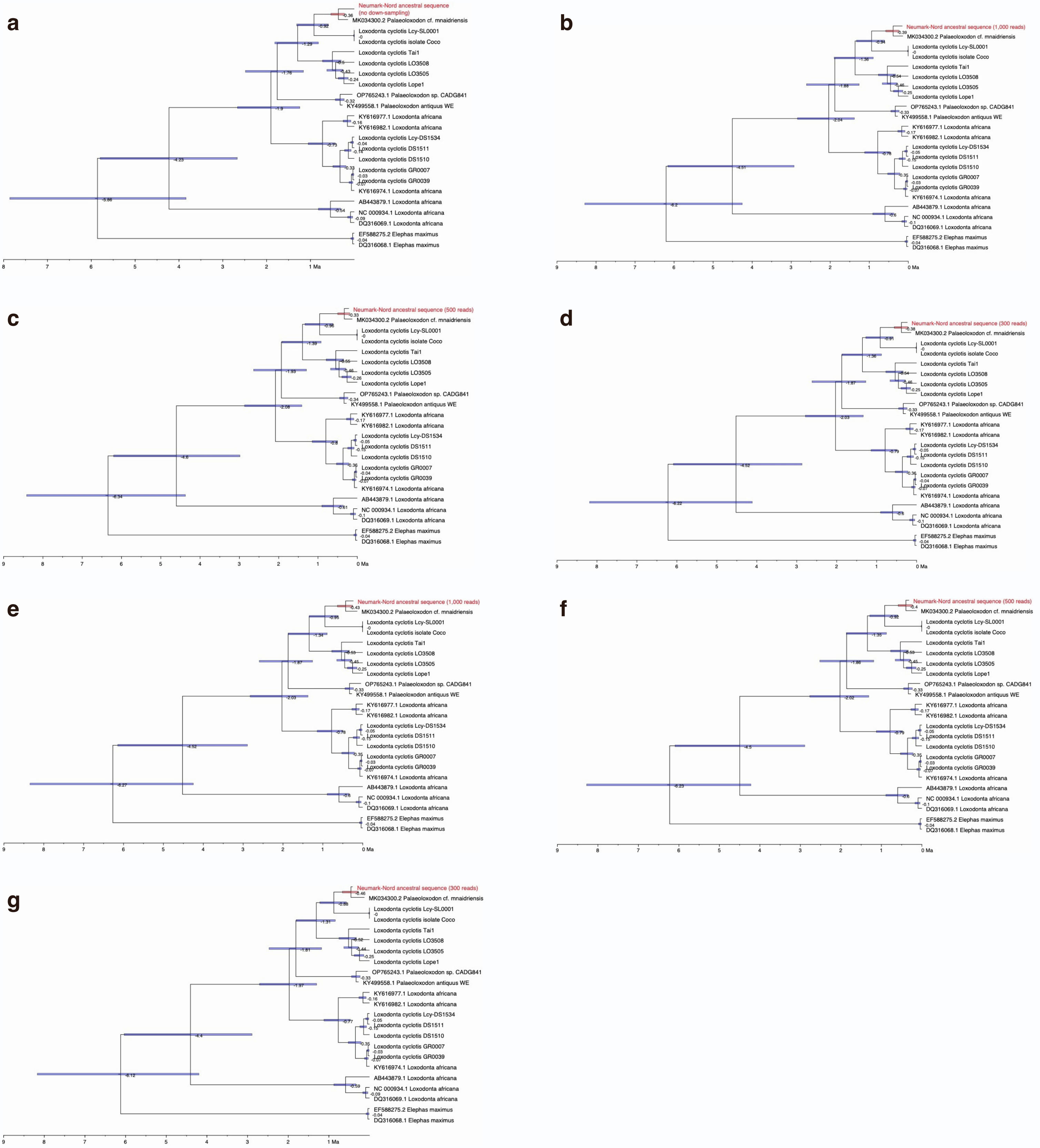

Figure S13. Effect of a high sequence error rate on time estimation. Ancestral sequences were inferred based on simulated mitochondrial genome sequences of two *P. antiquus* individuals from Neumark-Nord (NEPEC and NEU2A), with a 1% sequencing error artificially introduced. Errors were introduced either into (**a**–**d**) consensus sequences or (**e**–**g**) raw NGS reads prior to consensus sequence construction. Specifically: (**a**) without down-sampling; (**b**, **e**) down-sampling to 1,000 reads; (**c**, **f**) down-sampling to 500 reads; and (**d**, **g**) down-sampling to 300 reads. Coalescent times were estimated using BEAST, with sequences divided into five partitions: 13 protein-coding genes (further divided by codon loci, with ND6 analyzed on the complementary strand), RNA genes (two rRNAs and 22 tRNAs, all converted to H-strand sequences), and the D-loop. The HKY+I+Γ model and a relaxed log-normal clock model were applied to each partition. Branch lengths in the phylogenetic tree represent estimated divergence times, with nodal values indicating time estimates (Ma) and nodal bars showing their 95% highest posterior density (HPD). Nodal bars representing the tMRCA of the NN clade, corresponding to the divergence time between the Sicilian and Neumark-Nord individuals, are highlighted in red. The two Neumark-Nord individuals are highlighted in bold red. The introduction of a high sequence error rate had minimal effect on the posterior probability distributions.

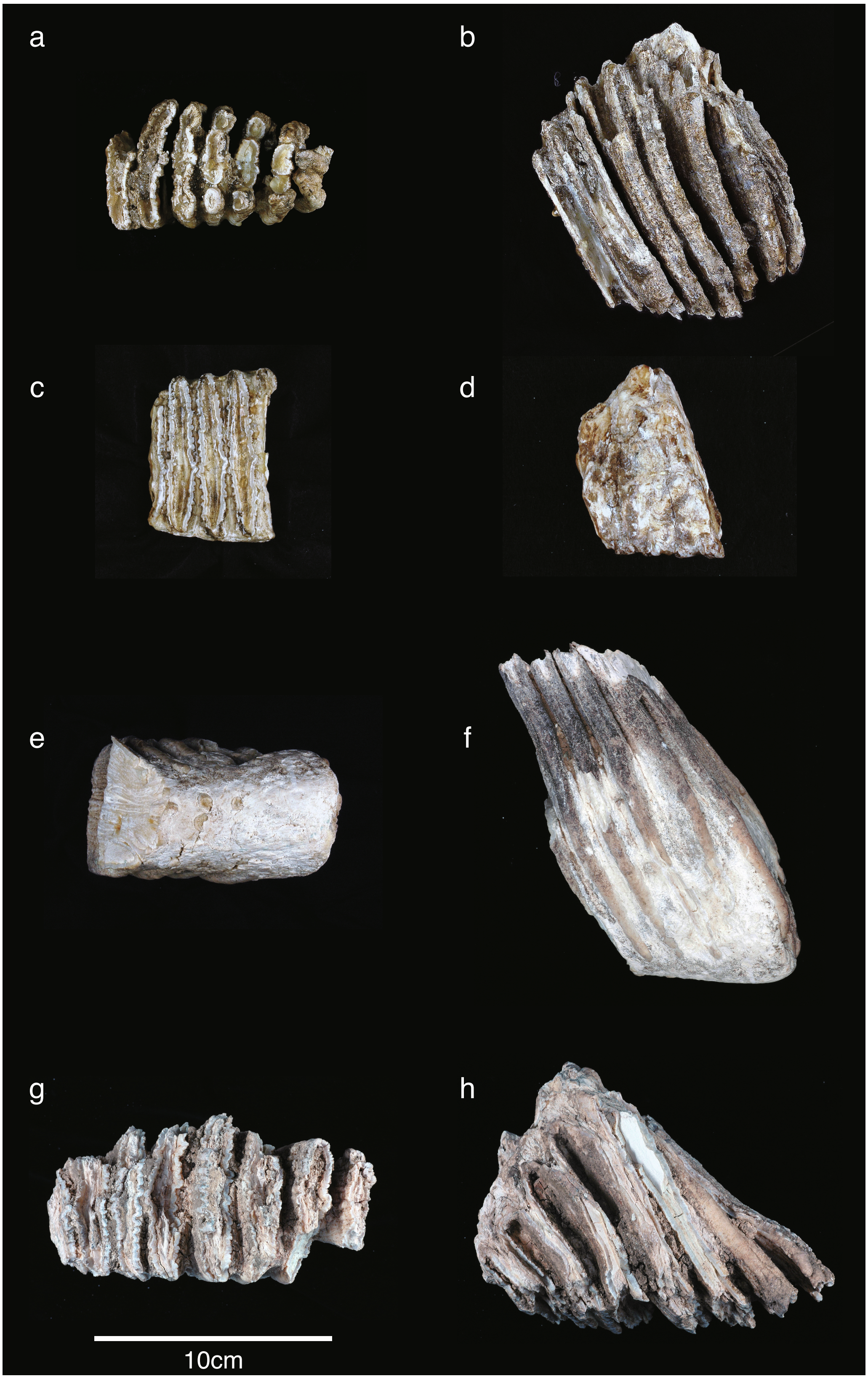

Figure S14. Molars of the extinct Japanese elephant, *Palaeoloxodon naumanni* (Makiyama, 1924 [S1]), excavated from the Upper Pleistocene cave deposits, Aomori Prefecture, northern Honshu, Japan.

a–b, AoPM 1975-5, left upper second molar; **c–d**, AoPM 1975-7, left upper second molar; **e–f**, NMNS-PV 22669, right upper second or third molar; **g–h**, NMNS-PV 22670, right upper second or third molar.

a, c, e, g, occlusal views; **b, d,** buccal views; **f, h,** lingual views. Anterior to the left. The scale bar, 10 cm.

Table S1. Sample information used for DNA analysis.

Individual Number	DNA extraction	Library construction	Raw reads	High quality reads	Mitochondria reads	Mitochondria read clusters
NMNS-PV 22669	Silica pellets	double strand	861,499,654	831,755,536	139	34
	Silica pellets	single strand	151,245,334	150,122,897	1	
	Silica column	single strand	147,038,796	144,627,649	2	
NMNS-PV 22670	Silica pellets	double strand	902,038,125	884,974,739	447	7,770
	Silica column	double strand	65,189,250	62,152,777	10	
	Silica pellets	single strand	216,207,056	163,354,327	207	
	Silica column	single strand	173,496,664	97,346,515	93	
	Silica pellets	baits	333,769,946	288,697,795	83,819,166	
AoPM1975-5	Silica pellets	double strand	41,941,626	41,744,257	5	12
	Silica column	double strand	36,871,533	35,141,286	2	
	Silica pellets	single strand	33,703,936	25,872,245	34	
	Silica column	single strand	40,760,606	26,661,078	21	
AoPM1975-7	Silica pellets	double strand	35,549,497	35,234,272	4	6,137
	Silica column	double strand	35,136,261	33,347,579	7	
	Silica pellets	single strand	1,085,178,636	841,149,395	662	
	Silica column	single strand	30,883,082	19,109,913	14	
	Silica pellets	baits	295,714,199	249,206,665	59,479,323	
			4,486,224,201	3,930,498,925	143,300,137	13,953

Table S2. Statistical analysis of the phylogenetic position of *P. naumanni* within and around the Eurasian-wide WE clade.

	Data1 (NMNS-PV22670 + AoPM1975-7 clade)				Data2 (NMNS-PV22670 only)				Data3 (AoPM1975-7 only)			
Topology[1]	lnL[2]	KH test[3]	SH test[4]	Bootstrap[5]	lnL[2]	KH test[3]	SH test[4]	Bootstrap[5]	lnL[2]	KH test[3]	SH test[4]	Bootstrap[5]
Tree1	<-34167.897>	-	-	100	<-33708.577>	-	-	100	<-33639.184>	-	-	100
Tree2	-81.993±17.876	0.000	0.005	0	-64.232±15.123	0.000	0.011	0	-68.094±16.107	0.000	0.008	0
Tree3	-82.036±17.852	0.000	0.005	0	-64.232±15.123	0.000	0.011	0	-68.079±16.120	0.000	0.008	0
Tree4	-120.486±22.026	0.000	0.000	0	-90.782±18.548	0.000	0.001	0	-90.216±18.436	0.000	0.001	0
Tree5	-120.486±22.026	0.000	0.000	0	-90.782±18.548	0.000	0.001	0	-90.216±18.436	0.000	0.001	0

[1] Tree topologies are shown in Figure S7.

[2] lnL(log likelihood) scores. Absolute values of lnL are shown only for the maximum likelihood phylogenetic trees (in angle brackets). For alternative phylogenetic trees, differences in lnL values relative to the ML tree are shown, along with standard deviations.

[3] P-values from the Kishino–Hasegawa (1989) test [S2].

[4] P-values from the Shimodaira–Hasegawa (1999) test [S4].

[5] Bootstrap probabilities as inferred by the resampling estimated log-likelihoods method (Kishino et al. 1990) [S3].

Likelihood estimation and statistical tests were performed using the BASEML program implemented in PAML. The mitochondrial genome was partitioned into five regions: first, second, and third codon positions of 13 protein-coding genes; RNA regions (2 rRNAs and 22 tRNAs); and the D-loop. Likelihoods were estimated under the GTR+Γ model accounting for differences in evolutionary rates, base composition, and substitution patterns across regions. For genes encoded on the L-strand, such as ND6, sequences were converted to their complementary strand equivalents. For Data1, the phylogenetic position of the *P. naumanni* clade was evaluated under the assumption of monophyly between the two individuals. For Data2 and Data3, phylogenetic inference was conducted based on each individual separately.

Supplemental References

[S1] Makiyama J (1924). Notes on a fossil elephant from Sahamma, Tôtômi. Memoir of the College of Science, Kyoto Imperial University, series B 1, 225-264.

[S2] Kishino H, Hasegawa M (1989). Evaluation of the maximum likelihood estimate of the evolutionary tree topologies from DNA sequence data, and the branching order in Hominoidea, J Mol Evol. *29*, 170-179.

[S3] Kishino H, Miyata T, Hasegawa M (1990). Maximum-likelihood inference of protein phylogeny and the origin of chloroplasts, J Mol Evol. *31*, 151-160.

[S4] Shimodaira H, Hasegawa M (1999). Multiple comparisons of log-likelihoods with applications to phylogenetic inference, Mol Biol Evol. *16*, 1114-1116.
